# Combined inhibition of PLK4 and PPM1D promotes cell death in cancer cells with 17q amplification

**DOI:** 10.1186/s12935-026-04391-3

**Published:** 2026-06-18

**Authors:** Miroslav Stoyanov, Cecilia Aquino-Perez, Shreyasi Mazumder, Dominika Salcakova, Silvia Petrezselyova, Libor Macurek

**Affiliations:** Cancer Cell Biology, Institute of Molecular Genetics, Czech Academy of Sciences, Prague, CZ-14220 Czech Republic

**Keywords:** PPM1D, PLK4, Breast cancer, Neuroblastoma, 17q amplification, Synthetic lethality

## Abstract

**Background:**

Polo-like kinase 4 (PLK4) is a key regulator of centriole duplication and contributes to mitotic spindle organization during cell division. PLK4 has been implicated as pharmacological target in a subset of breast cancers and neuroblastomas harboring 17q chromosomal gains and overexpression of the E3 ubiquitin-protein ligase TRIM37. Here, we investigated factors that influence sensitivity of cancer cells to PLK4 inhibition.

**Methods:**

We used cBio Cancer Genomics Portal and DepMap portal and performed bioinformatic analysis of copy number variations and gene expression to identify potential pharmacologically targetable genes in invasive breast carcinoma with 17q gains. Subsequently, we tested sensitivity of cancer cells to combinations of PLK4 inhibitor with other small molecule compounds and determined the impact on cell proliferation, cell death and gene expression. Validation was performed using a chemically unrelated PLK4 inhibitor, by RNAi-mediated depletion of PLK4 and in *TP53* knock-out cells.

**Results:**

We show that sensitivity of breast cancer cells with 17q23 amplification to PLK4 inhibitor is limited by the activity of protein phosphatase PPM1D, an established negative regulator of the tumor suppressor p53. Combined inhibition of PLK4 and PPM1D stimulates the expression of p53 target genes and promotes apoptosis in breast cancer cells with 17q gains. Similarly, combined inhibition of MDM2 and PLK4 was synthetically lethal in breast cancer cells with 17q gains, whereas loss of p53 suppressed the cell death induced by PLK4 inhibition. Finally, we show that PLK4 and PPM1D or MDM2 are synthetically lethal targets in IMR-32 and CHP-134 neuroblastoma cells, confirming that targeting negative regulators of p53 enhances the efficiency of the PLK4 inhibitor in various cancers with 17q amplification.

**Conclusions:**

Our findings demonstrate that the combined inhibition of PLK4 and PPM1D or MDM2 sensitizes breast cancers and neuroblastoma with 17q gains and provide a rationale for advancing this novel drug combination into future preclinical development.

**Supplementary Information:**

The online version contains supplementary material available at https://doi.org/10.1186/s12935-026-04391-3.

## Introduction

Breast cancer is the most frequent cancer type in female population worldwide, and despite the advances in treatment, it remains a major cause of death. Personalized treatment strategies are therefore needed to efficiently target specific cancer types. Perhaps, one of the best examples of personalized treatments is the use of PARP inhibitors, which selectively target cancers with deficient homologous recombination due to the inactivating mutations in *BRCA1* and *BRCA2* genes [1]. Understanding the genetic background of tumors may thus help to identify potential vulnerabilities of cancer cells. Comparative genomic hybridization (CGH) has revealed that amplification of the 17q22-24 genomic region is common in breast cancer, and several genes within this region have been proposed to act as oncogenes [2–7]. A large analysis of copy number variants and single nucleotide polymorphisms in approx. 2,000 primary breast cancers revealed that tumors harboring the 17q23 amplification (classified as integrative cluster 1) comprise mostly of ER-positive, luminal B tumors with a 10-year relapse-free survival 64% [8, 9]. Among the genes in the 17q23 locus, amplification of *PPM1D* (encoding Protein Phosphatase Magnesium-Dependent 1 Delta, also known as wild type p53-induced phosphatase 1 (WIP1)) has been shown to promote breast cancer development by suppressing p53 and p38/MAPK pathways [10–13]. Conversely, loss of *PPM1D* in mouse models blocked development of the *MMTV*-ErbB2-driven mammary tumorigenesis as well as growth of the myc-induced lymphomas and APC^min^-induced colon cancer [10–15]. Similarly, a small molecule inhibitor of PPM1D induced cell death and senescence in the breast cancer cells with high PPM1D levels and intact p53 pathway, while healthy cells were relatively insensitive to PPM1D inhibition [16, 17]. Besides breast cancer, PPM1D has been proposed as an attractive pharmacological target in hematological malignancies, and intensive development of PPM1D inhibitors is currently ongoing [16, 18, 19].

Polo-like kinase 4 (PLK4) is a highly conserved serine/threonine kinase essential for correct centriole duplication during the S phase of the cell cycle, which allows formation of a bipolar mitotic spindle in mitosis [20, 21]. PLK4 is recruited to the centriole by proteins Cep192 and Cep152, and its activity is needed for the binding of the STIL/SAS6 complex and subsequent assembly of the cartwheel structure during centriole duplication [22, 23]. In the non-transformed cells, centrosome loss caused by PLK4 inhibition prolongs the duration of acentrosomal cell division and results in cell cycle arrest in the subsequent G1 phase [24–26]. The cell cycle arrest caused by PLK4 inhibition depends on the mitotic surveillance pathway, involving USP28- and 53BP1-dependent activation of p53 [24–26]. Data from TCGA database show that PLK4 is overexpressed in multiple cancers, including breast invasive carcinoma [27]. Recently, it has been demonstrated that a subset of breast cancer cells with *TRIM37* amplification are hypersensitive to the loss of PLK4 activity, and PLK4 has been proposed as a potential pharmaceutical target in breast cancers with 17q23 amplification [28, 29]. TRIM37 is an E3 ubiquitin ligase that reduces microtubule nucleation in the pericentriolar matrix by promoting degradation of centrobin [30]. Increased levels of TRIM37 can therefore sensitize breast cancer cells to PLK4 inhibition by impairing bipolar mitotic spindle formation, leading to extensive chromosomal aberrations.

Because inhibition of PLK4 or PPM1D leads to activation of the p53 pathway, although through distinct molecular mechanisms, we hypothesized that the combined inhibition of these proteins may enhance the cellular response. We tested this hypothesis in breast cancer cell lines with amplification of the 17q22-24 locus, which carries the neighboring *PPM1D* and *TRIM37* genes. We found that inhibition of PPM1D using the validated small molecule inhibitor GSK2830371 increased the sensitivity of MCF7 and BT474 cells to the PLK4 inhibitor centrinone. In contrast, control T47D cells lacking the 17q amplification were not sensitive to either PPM1D or PLK4 inhibition. Furthermore, we found out that the combined inhibition of PLK4 and PPM1D enhanced p53 activation and induced apoptosis. Finally, we confirmed the synthetic lethal interaction of PLK4 and PPM1D inhibitors in IMR-32 and CHP-34 neuroblastoma cells that carry 17q gains. In summary, we propose that the cytotoxic effect of PLK4 inhibition in cancers with 17q amplification can be potentiated by concomitant inhibition of PPM1D.

## Methods

### Antibodies and reagents

Centrinone, ocifisertib (CFI-400945 [31]), GSK2830371 and Z-VAD-FMK were from MedChemExpress, were dissolved in DMSO and were used at indicated concentrations. The antibodies used in this study are as follows: Caspase 3 cleaved (Asp175) (clone 5A1E, 9664 S), Caspase 7 cleaved (Asp198) (clone D6H1, 8438 S), cleaved PARP (Asp214) (clone D64E10, 5625), PARP1 (clone 46D11, 9532), GAPDH (clone D16H11, 5174), KAP1-pS824 (#4127T), p53-pSer15 (82530), histone H3 (14269) from Cell Signaling Technology; p53 (sc-126), p21 (sc-6246), PPM1D (sc-376257), TFIIH (sc-293), importin β (sc-137016) from Santa Cruz Biotechnology; caspase 3 (19677-1-AP), caspase 7 (27155-1-AP), TRIM37 (13037-1-AP) from Proteintech; g-tubulin (clone GTU-88, T6557) and phospho-Histone H2A.X-Ser139 (05–636) from Sigma; α-tubulin (orb323092) from Biorbyt, Alexa Fluor-conjugated antibodies and TRIM37 (A301174AT) from Thermo Fisher Scientific and HRP-conjugated antibodies for immunoblotting from BioRad.

### Cells

MCF7 (RRID: CVCL_0031) and hTERT RPE-1 (RRID: CVCL_4388, hereafter referred to as RPE) cells were from ATCC. MCF7-TP53-KO cells lacking p53 were previously reported [17]. ZR-75-30 (RRID: CVCL_1661) and T47D (RRID: CVCL_0553) were obtained from European Collection of Cell Cultures, IMR-32 (RRID: CVCL_0346) and CHP-134 (RRID: CVCL_1124) from DSMZ-German Collection of Microorganisms and Cell Cultures. BT474 (RRID: CVCL_0179) were obtained from Jaroslav Truksa (Institute of Biotechnology, Vestec), MCF-10 A (RRID: CVCL_0598) from Claus Sørensen (University of Copenhagen, Denmark) and U2OS (RRID: CVCL_0042) from Rene Medema (Princess Máxima Center, Netherlands). Cells were routinely grown in high glucose DMEM supplemented with 10% FBS, Penicillin (100 U/ml) and Streptomycin (0.1 mg/ml). Alternatively, RPMI-1640 media supplemented with 20% FBS, glutamine and sodium pyruvate was used for ZR-75-30, IMR-32 and CHP-134 cells. MCF-10 A cells were grown in DMEM/F12 media supplemented with 5% horse serum, Penicillin (100 U/ml) and Streptomycin (0.1 mg/ml), insulin (10 µg/ml), hydrocortisone (0.5 µg/ml), EGF (20 ng/ml) and cholera toxin (100ng/ml, Sigma). RPE-PPM1D-KO and U2OS-PPM1D-KO cells that were used as negative controls were described previously [32]. All cell lines were regularly checked for absence of mycoplasma contamination using MycoAlert Plus reagent (Lonza). Control, PLK4 (targeting sequence GGACCUUAUUCACCAGUUA) or TRIM37 (targeting sequence UUGUUGGAUAUAACUAGUC) Silencer Select siRNA was used at final concentration 5 nM and was transfected using RNAiMAX reagent (Thermo Fisher Scientific).

### Cell viability assay

Cellular proliferation and viability were assayed as described previously [33]. Briefly, cells were seeded in 96-well plates (100 cells/well). Following day, cells were treated or not with indicated inhibitors and cultivated for 7 days. After exchanging the fresh media, cells were incubated with resazurin (30 µg/ml, Merck) at 37 °C for 3 h. Fluorescence at 560 nm excitation wavelength and 590 nm emission was measured using Envision plate reader (Perkin Elmer). Average signal from eight technical replicates was calculated and values were normalized to the non-treated condition. The experiment was performed in three biological replicates. To evaluate dose–response effects, MCF7 cells were seeded on a 96-well plate at a density of 500 cells/well and treated with PPM1Di (50–400 nM) and centrinone (25–300 nM) for 5 days and cell viability was monitored using resazurin as described above. The cell viability was calculated from ten replicates. Drug synergy was quantified using multiple synergy reference models (Bliss, Loewe, and Highest Single Agent/HSA) with the SynergyFinder3.0 web application [34].

### Clonogenic assay

MCF7 cells (1000/well) were on a 6-well plate and were grown in media containing either GSK2830371, centrinone or their combination for 2 weeks with replacing fresh media after each 4 days. Where indicated, cells were washed with PBS and cultivated for additional 2 weeks in fresh media. Cells were fixed with ethanol and stained with crystal violet solution for 15 min. The plates were then washed thoroughly with water and left to dry out. Automatic quantification of colonies was done in ImageJ using Analyze particles tool. Alternatively, the low adherent CHP-134 and IMR-32 cells were grown and treated on 10 cm plates for 4 days, were subsequently seeded on 6-well plates in the fresh media with indicated inhibitors and cultivated for additional 4 days before fixation.

### Determination of cell death

MCF7 cells grown on 10 cm plates were cultivated in media supplemented with indicated concentrations of GSK2830371, centrinone, or their combination for 5 days and were subsequently collected for either flow cytometry (FACS) or western blot analysis. For the FACS, the culture medium was collected in 10 ml tubes (to collect all dead cells), cells were trypsinized and collected. After washing twice with PBS, cells were fixed in 0.5 ml of 4% PFA and permeabilized with ice-cold methanol. Cells were stained with primary and secondary antibodies and analyzed by flow cytometry using LSR II cytometer (BD Biosciences) and FlowJo software. Single cells were gated on forward scatter area versus forward scatter height (FSC-A vs. FSC-H) to exclude doublets and aggregates and fraction of Cleaved Caspase-7 positive cells was determined. Alternatively, whole cell lysates (20 µg protein) were separated on gradient gels by SDS-PAGE and after western blotting, membranes were probed with cleaved caspase 3/7 or cleaved PARP antibodies. Staining of the same membranes for TFIIH, histone H3, or importin β was used as loading control.

### qRT-PCRc

DNA was generated from RNA using random hexamer primers and RevertAid H Minus Reverse Transcriptase (Thermo Scientific) following the manufacturer’s protocol. The following primers were used for *CDKN1A* (GGCGGCAGACCAGCATGACA and CCTCGCGCTTCCAGGACTGC), *NOXA* (GCTGGGGAGAAA-CAGTTCAG and AATGTGCTGAGTTGGCACTG), *PUMA* (TCTCGGTGCTCCTTCACTCT and ACGTTTGGCTCA-TTTGCTCT), *BAX* (GCTGGACATTGGACTTCCTC and GTCTTGGATCCAGCCCAAC), *TP53* (CAGCACATGACGG-AGGTTGT and TCATCCAAATACTCCACACGC), *PPM1D* (AAGGCTTTCTCGCTTGTCACC and TGAGTCAC-CTACGTGAGCTAC) and *GAPDH* (ggtctcctctgacttcaaca and gtgagggtct-ctctcttcct). qRT-PCR was performed using FastStart DNA Master SYBR Green I and LightCycler 480 II (Roche). The analysis was done using the ΔΔCT method and normalized to the *GAPDH* expression level.

### Data analysis

Copy number variations and mutations of *PPM1D*,* TRIM37* and *TP53* genes were evaluated in a set of invasive breast carcinoma samples (*n* = 9553) using the cBio Cancer Genomics Portal (http://cbioportal.org) [35]. Pairwise combinations of the genetic variations were analyzed for co-occurrence or mutual exclusivity in the queried samples and calculated p value < 0.05 was considered significant. Expression of *PPM1D* and *TRIM37* was evaluated in 63 invasive breast cancer cell lines using Data Explorer 2.0 tool in DepMap portal (https://depmap.org/portal/) [36]. Values were inferred from the RNA-seq data using RSEM (unstranded mode), and were reported after log2 transformation, using a pseudo-count of 1; log2(TPM + 1).

## Results

### Breast cancer cells are highly sensitive to combined treatment with PLK4 and PPM1D inhibitor

**Fig. 1:**
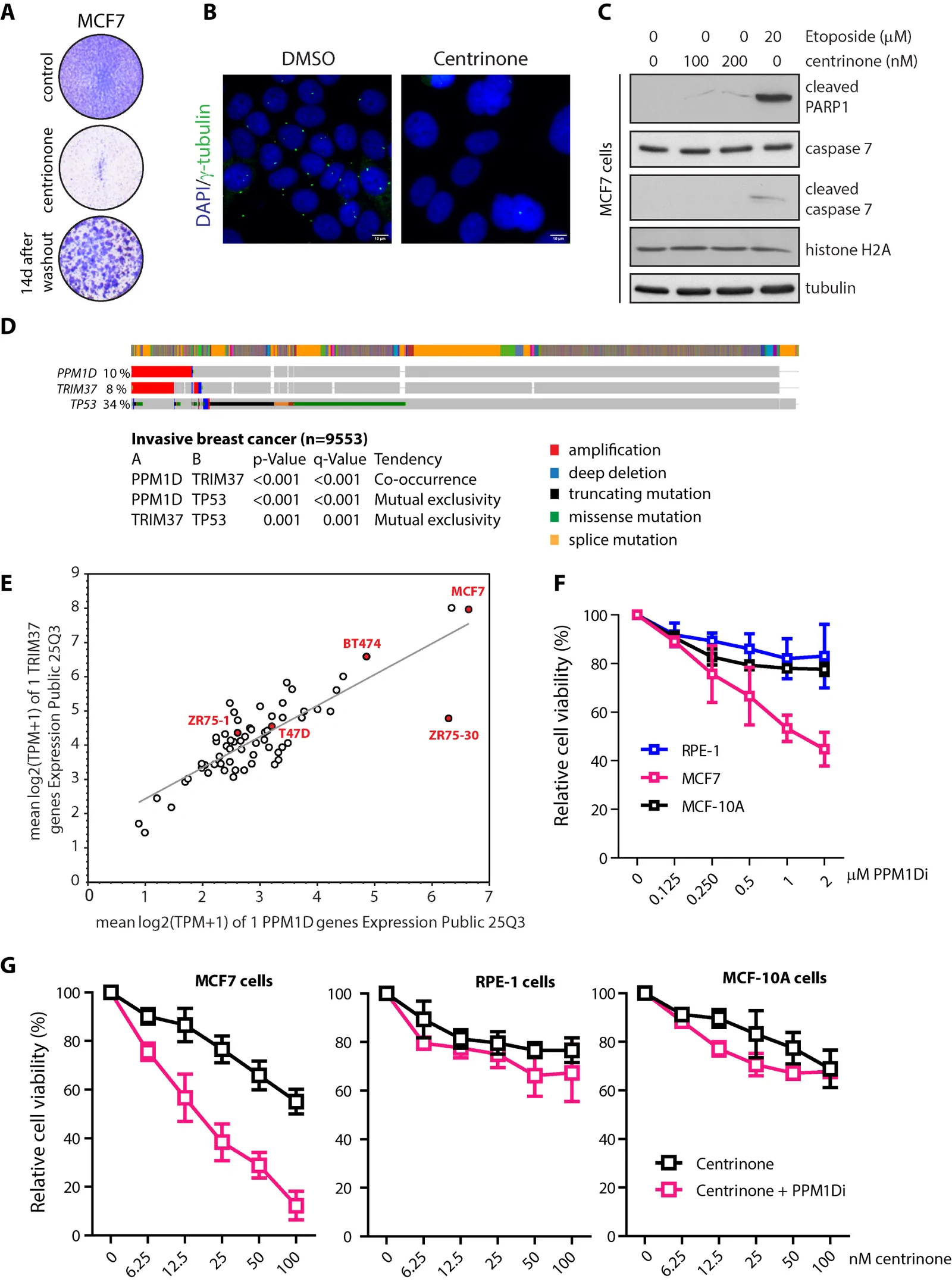
MCF7 breast cancer cells are sensitive to the combined inhibition of PLK4 and PPM1D (**A**) MCF7 cells seeded on 6-well plates were treated or not with centrinone (100 nM) for 2 weeks, fixed with ethanol and stained with crystal violet. Alternatively, cells treated with centrinone were washed with PBS and grown in fresh media for another 2 weeks. Representative images are shown. (**B**) MCF7 cells were treated with DMSO or centrinone (100 nM) for 10 days, fixed and stained for the centrosome marker g-tubulin and DAPI. Representative image is shown, bar represents 10 mm. Note the absence of g-tubulin staining in centrinone treated cells and enlargement of cell nuclei. C) MCF7 cells were treated with DMSO, centrinone (100 or 200 nM) or etoposide (20 mM) for 10 days, and induction of cell death was assayed in whole cell lysates by probing for cleaved caspase 7 and cleaved PARP. (**D**) *PPM1D*, *TRIM37* and *TP53* genes were scored for copy number variations and mutations in 9,553 samples of the invasive breast carcinoma available from cBioPortal portal. Colors indicate various types of genetic variations. Co-occurrence or mutual exclusivity was calculated by the tool at cBioPortal portal and p<0.05 was considered statistically significant. (**E**) Expression of *PPM1D* and *TRIM37* genes was evaluated in 63 cell lines derived from invasive breast cancer using Data Explorer tool in DepMap portal. (**F**) Sensitivity of RPE-1, MCF10A and MCF7 cells to indicated doses of PPM1D inhibitor was compared by resazurin assay. Normalization was performed to non-treated conditions for each cell type (n=3).(**G**) Survival of RPE-1, MCF10A and MCF7 cells treated with indicated doses of centrinone or combination of centrinone with PPM1D inhibitor (250 nM) using resazurin assay. Normalization was performed to non-treated conditions for each cell type (n=3)

Breast cancer cells carrying amplification of the genomic locus 17q22-24 have recently been reported to be highly sensitive to PLK4 inhibition by centrinone [28, 29]. In agreement with this report, we observed a strongly reduced proliferation of MCF7 cells after two weeks of treatment with 100 nM centrinone, a dose that induces the centriole loss, as visualized by the absence of γ-tubulin staining (Fig. 1 A and B). Nevertheless, after removal of centrinone, proliferation of the surviving cells was renewed, and eventually these cells fully recovered (Fig. 1 A). This suggests that while PLK4 inhibition efficiently suppresses cellular proliferation, it may not be sufficient to efficiently eliminate cancer cells. Consistent with this possibility, we observed only a low level of cleaved PARP and cleaved caspase 7 in MCF7 cells treated with centrinone compared to cells treated with the DNA damage-inducing drug etoposide (Fig. 1 C). Overexpression of TRIM37 has previously been shown to mediate the sensitivity of breast cancer cells with 17q22-24 amplification to centrinone [28, 29]. Inspired by this finding, we asked if other genes in the amplified region could also influence sensitivity to PLK4 inhibition. Amplification of the *PPM1D* gene was originally reported in about 10% of breast cancers that preferentially retain the wild type *TP53* [12], and this observation has been confirmed by a large dataset of invasive breast cancer samples available in the cBioPortal database (Fig. 1D). Interestingly, the percentage of *PPM1D* and *TRIM37* amplification is comparable (10% of *PPM1D* and 8% of *TRIM37*) and their expression levels are highly correlated (Pearson correlation = 0.830, Spearman correlation 0.726, *p* = 3.94E-17) across 63 invasive breast cancer cell lines, suggesting that these neighboring genomic loci are commonly co-amplified (Fig. 1D and E). We and others have shown that elevated levels of PPM1D can suppress activation of the p53 pathway, whereas inhibition of PPM1D can promote p53-dependent cell death or senescence [17, 32, 37]. Although GSK2830371 (hereafter referred to as the PPM1D inhibitor [16]), efficiently suppresses survival of MCF7 cells at higher doses [17], treatment with 250 nM dose resulted only in a modest reduction in cell proliferation (Fig. 1 F). As expected, the control non-transformed RPE-1 and MCF-10 A cells, which express low levels of PPM1D, were relatively insensitive to PPM1D inhibition (Fig. 1 F, Suppl. Figure 1 A) [17]. Importantly, we observed a significantly reduced survival of MCF7 cells by the combined treatment with PPM1D inhibitor and centrinone (Fig. 1G). In contrast, control RPE-1 and MCF-10 A cells were not further sensitized by the combination of PPM1D inhibitor with centrinone (Fig. 1G). Similarly, we observed that loss of PPM1D did not sensitize RPE-PPM1D-KO cells to centrinone (Suppl. Figure 1B). Finally, we tested the effect of centrinone on sensitivity of U2OS cells that contain high levels of active PPM1D due to the gain-of-function mutation in the last exon of *PPM1D* gene but do not show amplification of the 17q locus [38]. We found that U2OS cells are not sensitive to centrinone, either alone or in combination with the PPM1D inhibitor or PPM1D deletion, as indicated by the low level of cleaved caspase 3 (Suppl Fig. 1 C). At the same time, we found that depletion of TRIM37 reduced the sensitivity of MCF7 cells to the combined inhibition of PLK4 and PPM1D, which is consistent with the expected mode of action of centrinone (Suppl Fig. 1D) [26, 29]. Overall, we conclude that breast cancer cells expressing high levels of PPM1D due to the amplification of the 17q23 locus are highly sensitive to the combined inhibition of PLK4 and PPM1D.

## Combined inhibition of PLK4 and PPM1D induces p53-dependent apoptosis

**Fig. 2:**
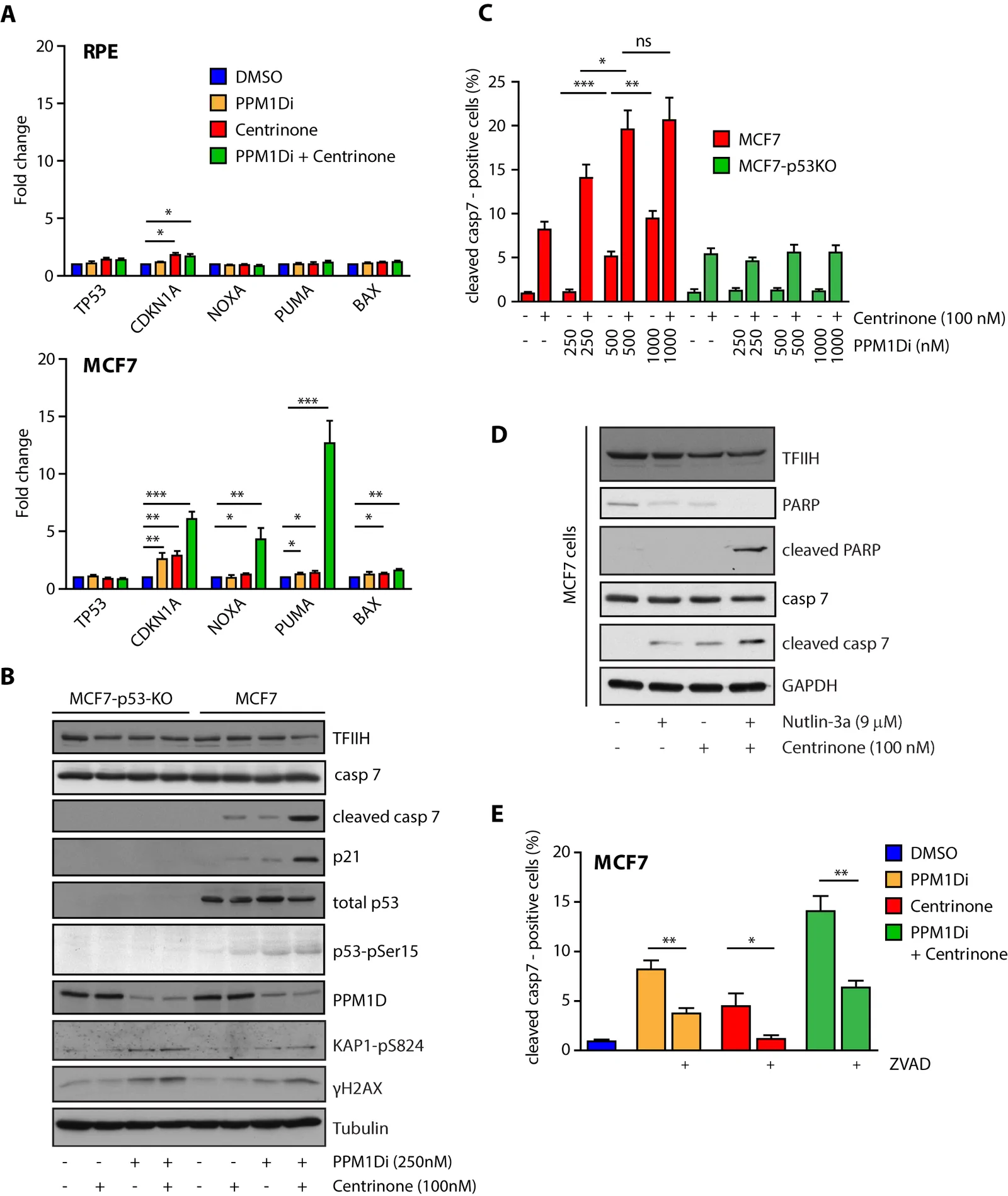
Combined inhibition of PLK4 and PPM1D induces apoptosis in breast cancer cells (**A**) MCF7 or RPE-1 cells were treated with centrinone (100 nM), PPM1Di (250 nM) or combination of both for 5 days and total cell RNA was collected. Expression of the indicated genes was determined by qRT-PCR. Levels were normalized to *GAPDH* expression and are presented as fold change to non-treated condition (n=3). Error bars indicate SDs, statistical significance was evaluated by Student's t-test, * p<0.05, ** p<0.01; *** p<0.001. (**B**) Whole cell lysates from MCF7 or MCF7-TP53-KO cells treated with centrinone (100 nM), PPM1Di (250 nM) or combination of both for 5 days were probed with indicated antibodies. A representative experiment from three biological replicates is shown. (**C**) MCF7 or MCF7-TP53-KO cells were treated with centrinone (100 nM), PPM1Di (250 - 1000 nM) or combination of both and were fixed after 5 days. Fraction of cleaved caspase 7 positive cells was determined by flow cytometry (n=3). Error bars indicate SDs, statistical significance was evaluated by Student's t-test; * p<0.05; ** p<0.01; *** p<0.001; ns, not-significant. (**D**) MCF7 cells were treated with centrinone (100 nM), nutlin-3 (9 mM) or combination of both for 5 days and whole cell lysates were probed with indicated antibodies. MCF7 cells were treated with centrinone (100 nM), PPM1Di (250 nM) or combination of both for 5 days. Z-VAD-FMK inhibitor was added where indicated. After fixation, fraction of cleaved caspase 7 positive cells was determined by flow cytometry (n=3). Error bars indicate SDs, statistical significance was evaluated by Student's t-test, * p<0.05, ** p<0.01

We next sought to determine the mechanism underlying the increased sensitivity of breast cancer cells to the combined inhibition of PLK4 and PPM1D. The centrosome loss following PLK4 inhibition is known to prolong the mitosis leading to activation of p53 through a USP28 and 53BP1-dependent mechanism [24–26]. PPM1D inhibition, on the other hand, can induce p53 response via multiple pathways, including stabilization of p53 levels by MDM2 inhibition, increased phosphorylation of S15, and indirect induction of p53 acetylation at K382 [39–41]. Indeed, we observed increased expression of p53 target genes, including *CDKN1A* after treatment of MCF7 cells with either centrinone or the PPM1D inhibitor (Fig. 2 A). Notably, *CDKN1A* expression further increased after the combined treatment with centrinone and PPM1D inhibitor (Fig. 2 A). Moreover, the combination of centrinone and PPM1D inhibitor efficiently induced expression of the two main p53 pro-apoptotic target genes *PUMA* and *NOXA* (Fig. 2 A). In contrast, RPE-1 cells did not show any significant induction of pro-apoptotic p53 target genes upon treatment with either centrinone, PPM1D inhibitor alone or in combination, and only mild upregulation of *CDKN1A* was observed. This corresponds well with the low effect of these treatments on RPE-1 cell proliferation (Fig. 2 A). Consistent with the induction of the pro-apoptotic genes, we detected cleaved caspase 7 in MCF7 cells treated with either centrinone or PPM1D inhibitor alone, and its level increased upon combined treatment (Fig. 2B). Similar results were detected by flow cytometry that detected an increasing fraction of cleaved caspase 7-positive cells with increasing doses of PPM1D inhibitor, and the fraction of dead cells was significantly increased by the combined treatment with centrinone (Fig. 2 C). Consistent with previous observations, we also observed that PPM1D inhibitor-induced cell death was fully dependent on the presence of p53 (Fig. 2 C). In contrast, we observed a small fraction of cleaved caspase 7-positive MCF7-TP53-KO cells in the presence of centrinone, although we note that PLK4 inhibition induced cell death much more effectively in cells with functional p53 pathway (Fig. 2 C). Further, we observed a strong induction of caspase 7 cleavage after combined treatment of MCF7 cells with centrinone and MDM2 inhibitor nutlin-3, supporting the importance of p53 function induction of cell death by PLK4 inhibition (Fig. 2D). In addition, the fraction of cleaved caspase 7-positive MCF7 cells was strongly reduced after treatment with the pan-caspase inhibitor Z-VAD-FMK, confirming that combined inhibition of PPM1D and PLK4 triggers apoptosis (Fig. 2E).

Finally, we evaluated the dose–response effects of centrinone and a PPM1D inhibitor in MCF7 cells using multiple synergy models implemented in SynergyFinder 3.0. We observed a high synergy score based on the Loewe additivity model (score = 13.588), whereas the Bliss independence model yielded a negative score (Suppl. Figure 2 A, 2B). This pattern is consistent with a model in which the cytotoxic effects of centrinone and the PPM1D inhibitor are mediated by a shared mechanism, such as the p53 pathway, which is activated by both compounds. Nevertheless, the high Loewe synergy score suggests a greater-than-additive effect of the combined treatment relative to the two individual compounds.

**Fig. 3:**
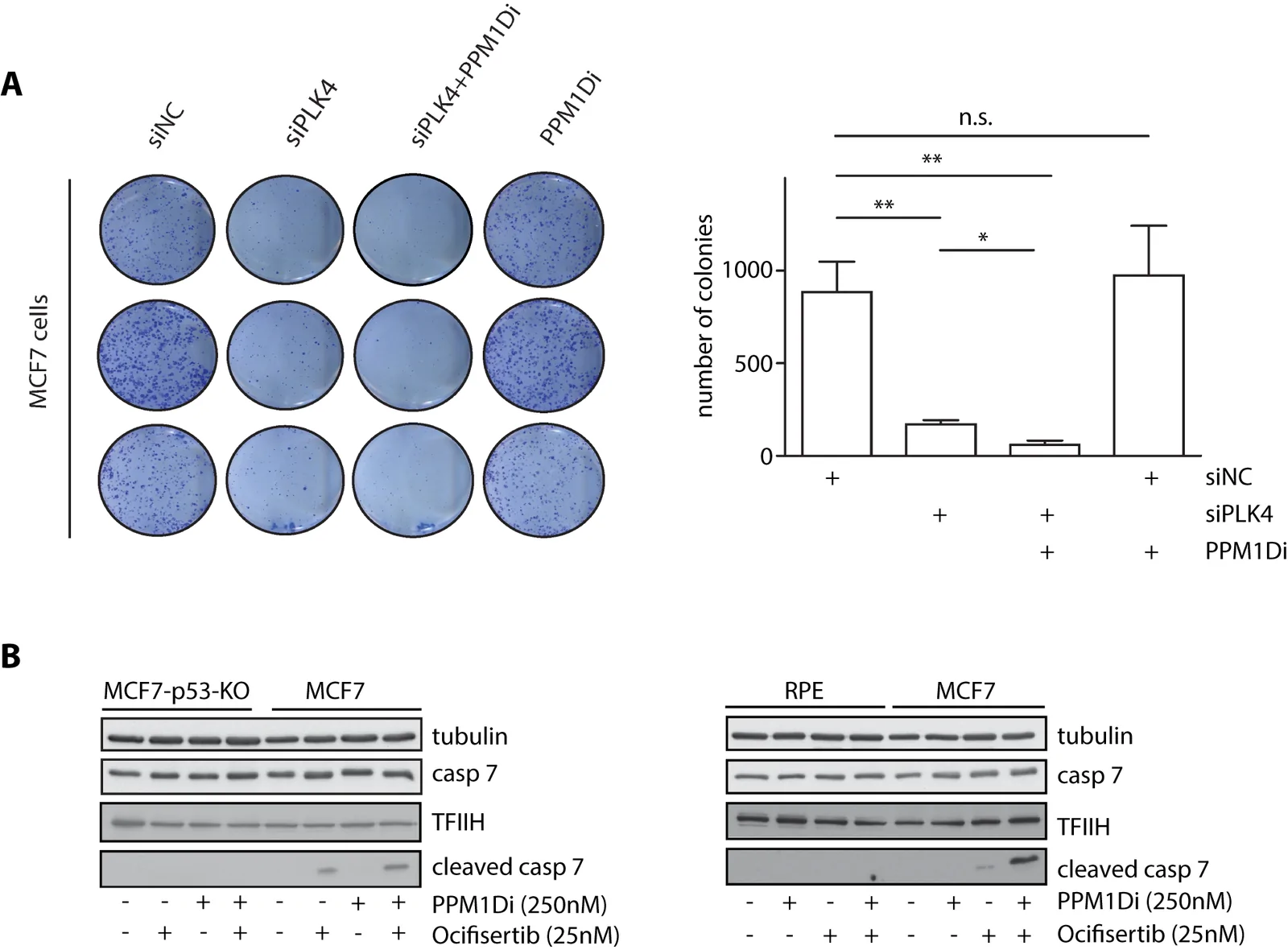
Increased cell death by combined inhibition of PPM1D and PLK4 depletion** (A**) MCF7 cells transfected with control or PLK4 siRNA were seeded (n=1000) on 6-well plates and were grown in the presence or absence of PPM1D inhibitor (250 nM) for 10 days. After fixation and staining with crystal violet, the number of colonies was quantified by Analyze particles tool in ImageJ (n=3). Statistical significance was evaluated by Student's t-test, ** p<0.01; ns, not-significant. (**B**) RPE-1, MCF7 and MCF7-TP53-KO cells were treated with ocifisertib (25 nM), PPM1Di (250 nM) or combination of both for 5 days and cleavage of caspase 7 was assayed by immunoblotting. Staining for TFIIH was used as a loading control. Representative images from two independent experiments are shown

To complement our results obtained by centrinone, we used RNA interference and showed that depletion of PLK4 strongly suppressed the colony formation in MCF7 cells. While treatment with a low concentration of the PPM1D inhibitor did not significantly affect the proliferation of control MCF7 cells, it further suppressed the colony formation in MCF7 cells depleted of PLK4 (Fig. 3 A). Finally, we aimed to confirm our observations using another PLK4 inhibitor, ocifisertib (25 nM [31]),, which is currently being tested in clinical trials for the treatment of AML/MDS and breast cancer [42, 43]. As expected, ocifisertib induced a basal level of caspase 7 cleavage in MCF7 cells, which was further increased by co-treatment with the PPM1D inhibitor (Fig. 3B). In contrast, neither control RPE-1 cells nor MCF7-TP53-KO cells showed caspase 7 activation after the combined treatment with ocifisertib and PPM1D inhibitor, which is consistent with a model in which the dual inhibition induces apoptosis in cancer cells in a p53-dependent manner (Fig. 3B).

### Cancer cells with 17q23 amplification are highly sensitive to the combined treatment with PLK4 and PPM1D inhibitor

**Fig. 4:**
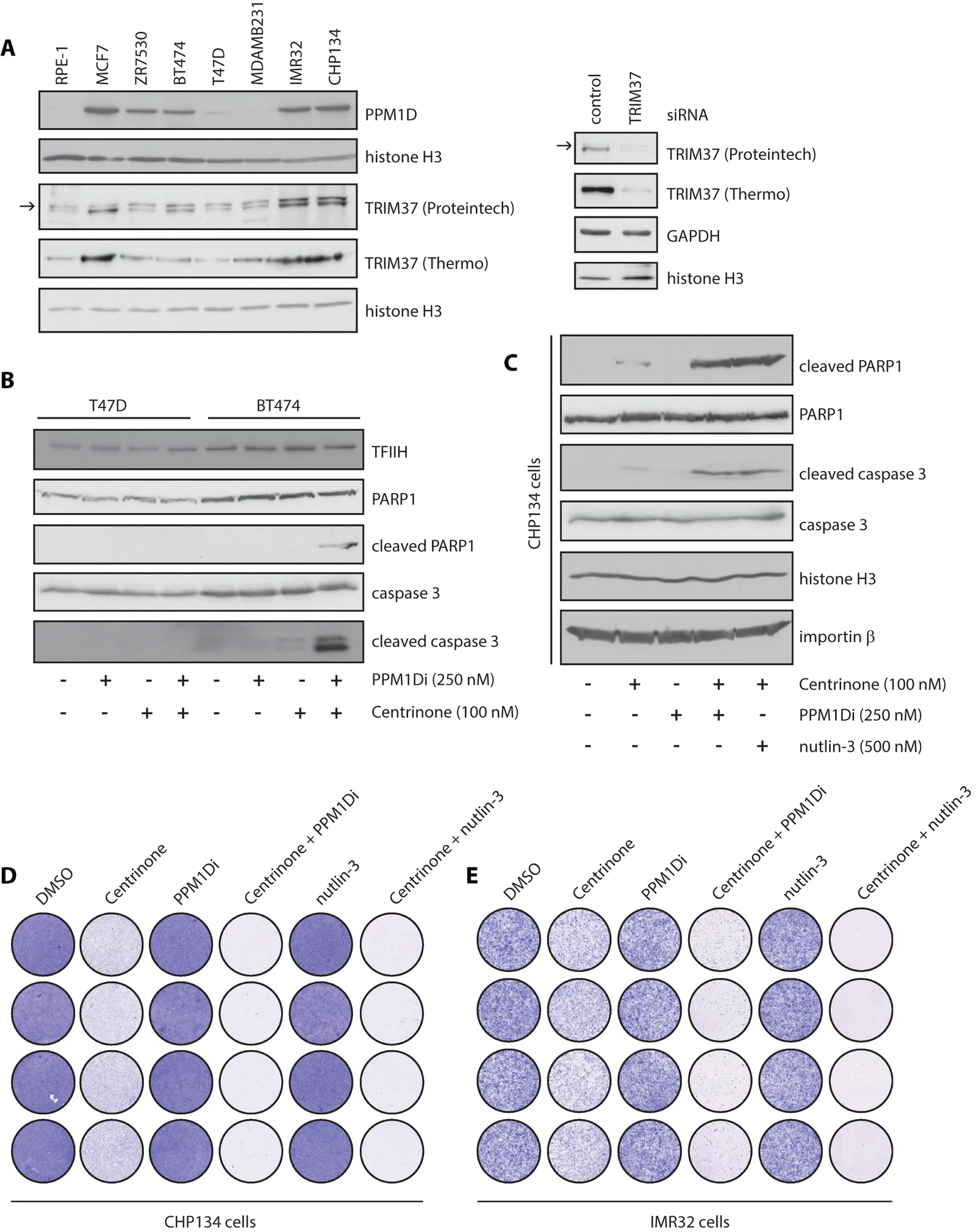
Combined PPM1D and PLK4 inhibition eliminates breast cancer or neuroblastoma cells with 17q gains (**A**) Comparison of PPM1D and TRIM37 levels in control RPE-1 and a panel of cancer cells. Staining for histone H3 was used as a loading control (left panel). Specificity of the two TRIM37 antibodies was determined by siRNA-mediated depletion of TRIM37 in MCF7 cells (right panel). Arrow indicates a cross-reacting band. (**B**) BT474 and T47D cells were treated with centrinone (100 nM), PPM1D inhibitor (250 nM) or combination of both for 5 days and whole cell lysates were probed for cleaved caspase 7 or cleaved PARP. TFIIH staining was used as a loading control. (**C**) CHP-134 cells were treated with centrinone (100 nM), PPM1D inhibitor (250 nM), nutlin-3 (500 nM) or indicated combinations for 5 days and whole cell lysates were probed for cleaved caspase 3 or cleaved PARP. Staining for importin b and 14-3-3 protein was used as loading control. (**D**) CHP-134 cells were treated with centrinone (100 nM), PPM1D inhibitor (250 nM), nutlin-3 (500 nM) or indicated combinations and grown for 8 days, fixed and stained with crystal violet. Representative images from four independent experiments are shown. (**E**) IMR-32 cells were treated with centrinone (50 nM), PPM1D inhibitor (250 nM), nutlin-3 (500 nM) or indicated combinations and grown for 8 days, fixed and stained with crystal violet. Representative images from four independent experiments are shown.

To further validate our findings, we assessed the sensitivity of breast cancer lines with different genetic backgrounds to PPM1D and PLK4 inhibitors. Similar to MCF7 cells, BT474 cells carrying amplification of the 17q23 locus and overexpressing PPM1D, were highly sensitive to the combined treatment PPM1D inhibitor and centrinone and showed high levels of cleaved caspase 3 and cleaved PARP (Fig. 4 A and B). In contrast, T47D cells, which express lower levels of PPM1D and TRIM37, were not sensitive to PLK4 inhibition, and also the combined inhibition of PPM1D failed to induce cleavage of caspase 3 and PARP indicative of the low level of cell death (Fig. 4 A and B). In addition to breast cancer, amplification of 17q has been described in a subset of neuroblastomas [44, 45]. Indeed, we observed high expression levels of PPM1D and TRIM37 in IMR-32 and CHP-134 cells (Fig. 4 A). As expected, we found that CHP-134 cells were sensitive to centrinone, and the combined treatment with centrinone and PPM1D inhibitor further increased the levels of cleaved PARP and caspase 3, indicative of enhanced cell death (Fig. 4 C). Proliferation of CHP-134 and IMR-32 cells was largely unaffected by a low dose of PPM1D inhibitor, but its combination with PLK4 inhibitor strongly suppressed cellular viability (Fig. 4D and E). Similarly, we found that proliferation of CHP-134 and IMR-32 cells was not significantly affected by a low dose of nutlin-3, but it greatly increased the sensitivity to the combined treatment with PLK4 inhibitor (Fig. 4D and E). We conclude that breast cancer and neuroblastoma cells harboring amplification of the 17q23 locus are highly sensitive to combined treatment with PPM1D and PLK4 inhibitors, as well as the dual inhibition of MDM2 and PLK4.

## Discussion

Increased PPM1D activity caused either by amplification of its genomic locus or by gain-of-function mutations in the last exon has previously been linked to the development of breast cancer, diffuse pontine glioma, neuroblastoma and hematological malignancies [44, 46–52]. In addition, increased PPM1D activity prevents cell death or senescence, thereby allowing cancer cells to escape the cytotoxic effects of chemotherapy [37, 51, 52]. In HER2 + breast cancer, amplification of the 17q23 locus and increased PPM1D activity have been linked with the resistance to trastuzumab [53]. In this study, we show that amplified *PPM1D* in breast cancer cells reduces the efficiency of PLK4 inhibition due to the partial suppression of p53 pathway. Conversely, inhibition of PPM1D in the breast cancer cells with 17q23 amplification enhances the cytotoxic effect of PLK4 inhibition by promoting expression of the p53 pro-apoptotic target genes *PUMA* and *NOXA*. Similarly, combined treatment with centrinone and the MDM2 inhibitor nutlin-3 increased cell death, further supporting the idea that induction of the p53 response may act synergistically with PLK4 inhibition. Of note, we did not observe any potentiation of cell death following the combined inhibition of PLK4 and PPM1D in p53-deficient cells. In summary, we propose that the combined inhibition of PPM1D and PLK4 can efficiently eradicate breast cancer cells with 17q amplification and retaining wild type p53. Beyond breast cancer, rearrangements of the 17q genomic region also occur in neuroblastoma, a common childhood cancer with poor prognosis [45]. Neuroblastoma cells with 17q gains have recently been shown to be sensitive to a novel PLK4 inhibitor RP-1664 and its efficiency was dependent on the presence of p53 [54–56]. In addition, PPM1D has previously been proposed as a potential target in neuroblastoma [57, 58]. Here, we show that the combined inhibition of PLK4 and PPM1D further sensitizes neuroblastoma cells, similarly to our observation in breast cancer cells, suggesting that this phenotype may be common for cancers with 17q gains.

PLK4 is an attractive therapeutic target, however, the clinical application of PLK4 inhibitors in cancer treatment has thus far been limited by the low bioavailability of centrinone and by the relatively low specificity of CFI-400,945, which targets not only PLK4 but with lower efficiency also Aurora B [54, 59]. Two new PLK4 inhibitors, RP-1664 and CZS-241, with improved pharmacological properties have recently entered clinical trials [54, 60]. Similarly, GSK2830371 is a potent PPM1D inhibitor in preclinical studies, but it lacks favorable pharmacokinetic properties for clinical use [16]. The development of next-generation PPM1D inhibitors derived from GSK2830371 with improved bioavailability provides hope for future use in cancer therapy [18, 52]. In addition, the chemically unrelated compound SL-176 is a potent PPM1D inhibitor that has recently been reported to synergistically suppress neuroblastoma growth in combination with GSK-J4, an inhibitor of the H3K27 demethylase JMJD3 [61, 62].

Here, we demonstrate an increased induction of cell death following combined inhibition of PPM1D and PLK4 in breast cancer and neuroblastoma cells carrying the wild type p53 and 17q gains. In these cancer types, we also observe a synthetic lethal interaction between PLK4 and MDM2, for which several small molecule inhibitors and degraders with favorable pharmacological properties are available. We propose that elevated PPM1D expression reduces the sensitivity of cancer cells to PLK4 inhibition, whereas combined treatment with MDM2 and PLK4 inhibitors allows efficient reactivation of p53, leading to eradication of cancer cells with 17q amplification. A limitation of this study is the lack of in vivo validation, as all findings are derived from in vitro cell line models; thus, the therapeutic efficacy and safety of combined PLK4 and PPM1D inhibition remain to be established in appropriate animal models, which will be an important focus of future studies.

## Supplementary Information


Supplementary Material 1.



Supplementary Material 2.



Supplementary Material 3.


## Data Availability

All data generated or analysed during this study are included in this published article. Reagents and materials generated in this study are available upon request.
