# Supplementary material for: Combined inhibition of PLK4 and PPM1D promotes cell death in cancer cells with 17q amplification
Source: Cancer Cell Int. 2026 Jun 18;26:298. doi: 10.1186/s12935-026-04391-3 (PMC13523465; doi:10.1186/s12935-026-04391-3)
Supplement: Supplementary file 1 [file 12935_2026_4391_MOESM1_ESM.pdf]

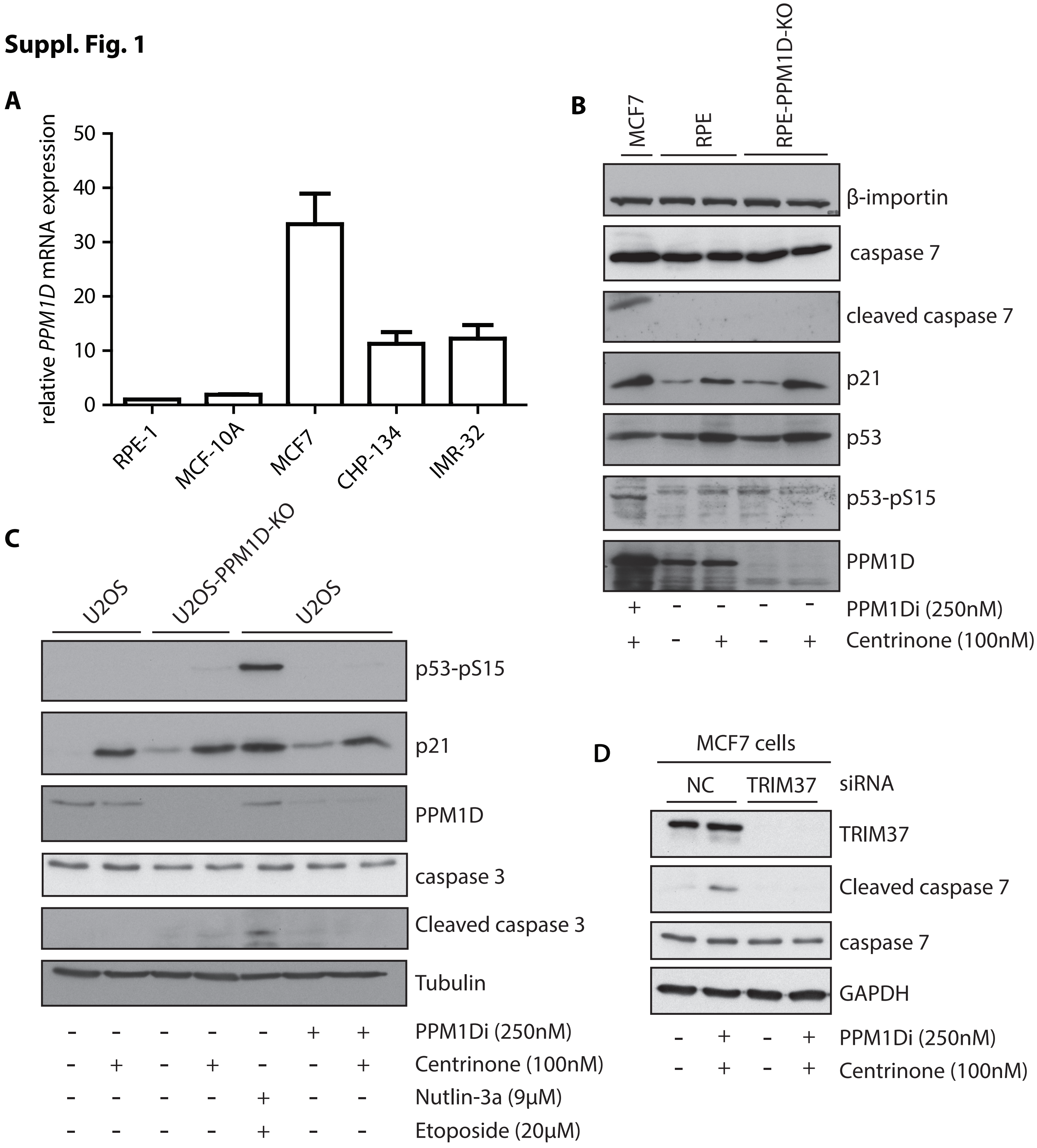

Supplementary Fig. 1. A) Expression of PPM1D in RPE-1, MCF-10A, MCF7, CHP134 and IMR-32 cells was determined by qRT-PCR. **B)** MCF7, RPE-1 and RPE-PPM1D-KO cells were treated with centrinone and PPM1D inhibitor for 5 days and whole cell lysates were probed with indicated antibodies. Note the absence of cleaved caspase 7 signal in RPE-1 and RPE-PPM1D-KO cells. MCF7 cells served as positive control. **C)** Parental U2OS or U2OS-PPM1D-KO cells were treated with centrinone and PPM1D inhibitor for 5 days and whole cell lysates were probed with indicated antibodies. Treatment with nutlin-3 and Etoposide was used as positive control for induction of cell death. **D)** MCF7 cells were transfected with control siRNA (NC) or siRNA targeting TRIM37 and were treated with combination of centrinone and PPM1D inhibitor for 5 days. Cleaved caspase 7 was used as marker of cell death.
