# Supplementary material for: Combined inhibition of PLK4 and PPM1D promotes cell death in cancer cells with 17q amplification
Source: Cancer Cell Int. 2026 Jun 18;26:298. doi: 10.1186/s12935-026-04391-3 (PMC13523465; doi:10.1186/s12935-026-04391-3)
Supplement: Supplementary file 2 [file 12935_2026_4391_MOESM2_ESM.pdf]

Suppl. Fig. 2

A

PPM1Di (nM) \ centrinone (nM)	0	25	50	100	150	200	250	300
400	26.7	19.1	19.9	19.3	19.6	19.2	19.8	13.0
350	48.8	62.4	42.2	26.0	27.7	26.9	26.2	22.1
300	52.5	52.5	33.3	21.9	26.0	25.2	23.5	24.4
250	57.8	58.4	33.3	25.0	26.9	29.1	26.6	22.1
200	65.3	71.6	45.4	27.9	29.6	29.3	27.6	25.0
100	78.2	84.5	49.7	28.2	30.4	33.8	30.3	27.4
50	75.6	95.8	57.4	33.7	38.7	34.1	33.5	27.9
0	100.0	59.9	38.2	36.5	35.7	33.5	30.3	26.0

inhib. (%)

B

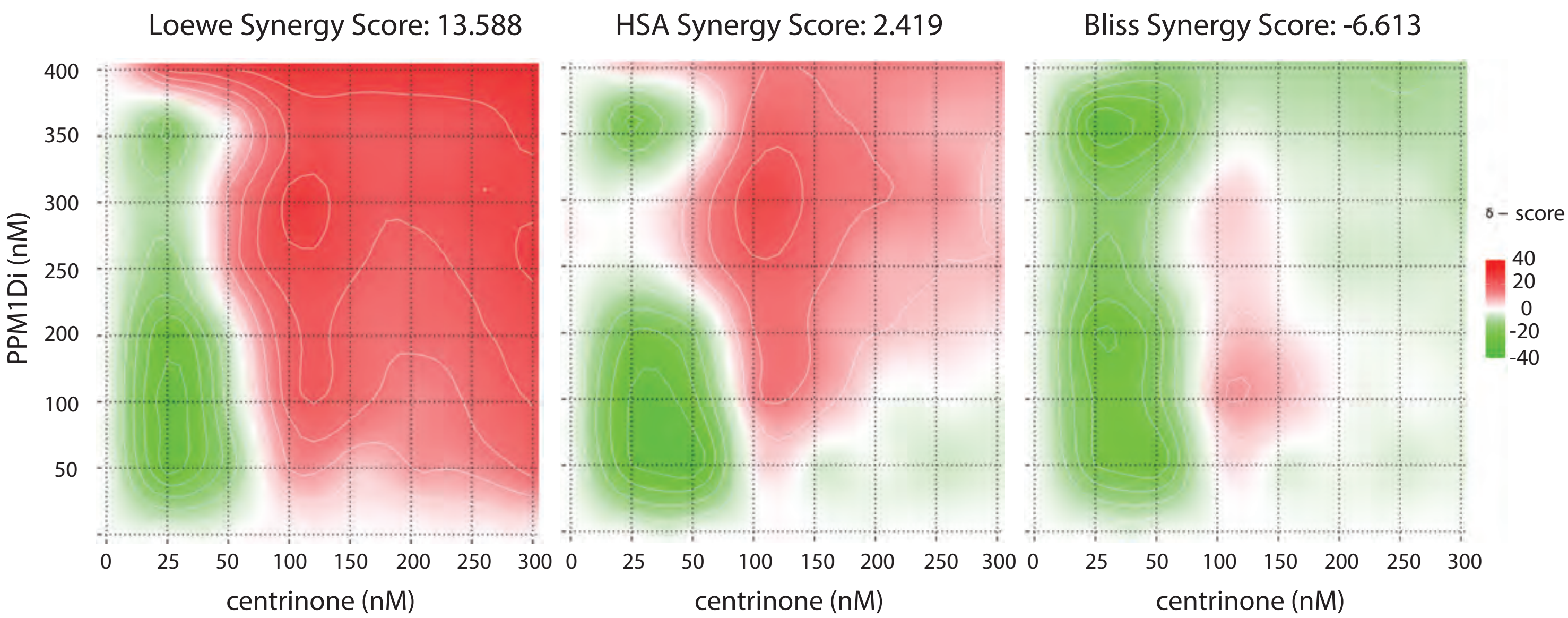

Supplementary Fig. 2. Dual inhibition of PPM1D and PLK4 synergistically impairs MCF7 cell growth. A) MCF7 cells were treated with increasing concentrations of the PPM1D inhibitor (PPM1Di; 50-400 nM) and the PLK4 inhibitor centrinone (25-300 nM). Cell viability was measured after 5 days of growth using staining with resazurin. The fluorescent signal was monitored using 560 nm excitation wavelength and 590 nm emission wavelength. Summary of the cell viability values (inhibition, %) of ten replicates, which was used for synergy modeling. **B)** Pharmacological interaction maps generated with SynergyFinder3.0 showing the combined effects of PPM1Di and centrinone.
