# Supplementary material for: Combined inhibition of PLK4 and PPM1D promotes cell death in cancer cells with 17q amplification
Source: Cancer Cell Int. 2026 Jun 18;26:298. doi: 10.1186/s12935-026-04391-3 (PMC13523465; doi:10.1186/s12935-026-04391-3)
Supplement: Supplementary file 3 [file 12935_2026_4391_MOESM3_ESM.pdf]

Fig. 1

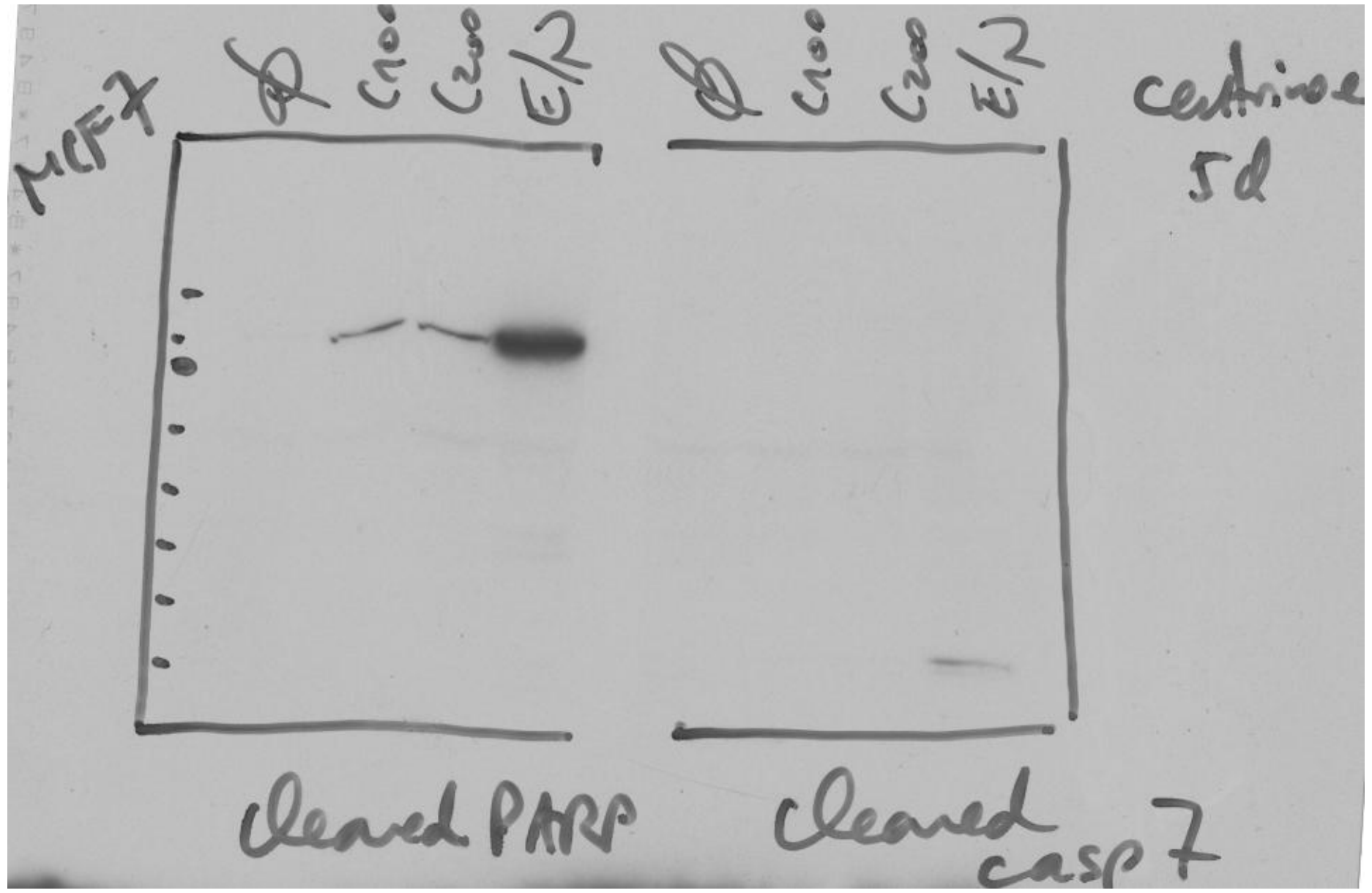
MCF7
Ø
C100
C200
E/2
Ø
C100
C200
E/2
centrinone
5d
cleaved PARP
cleaved casp7

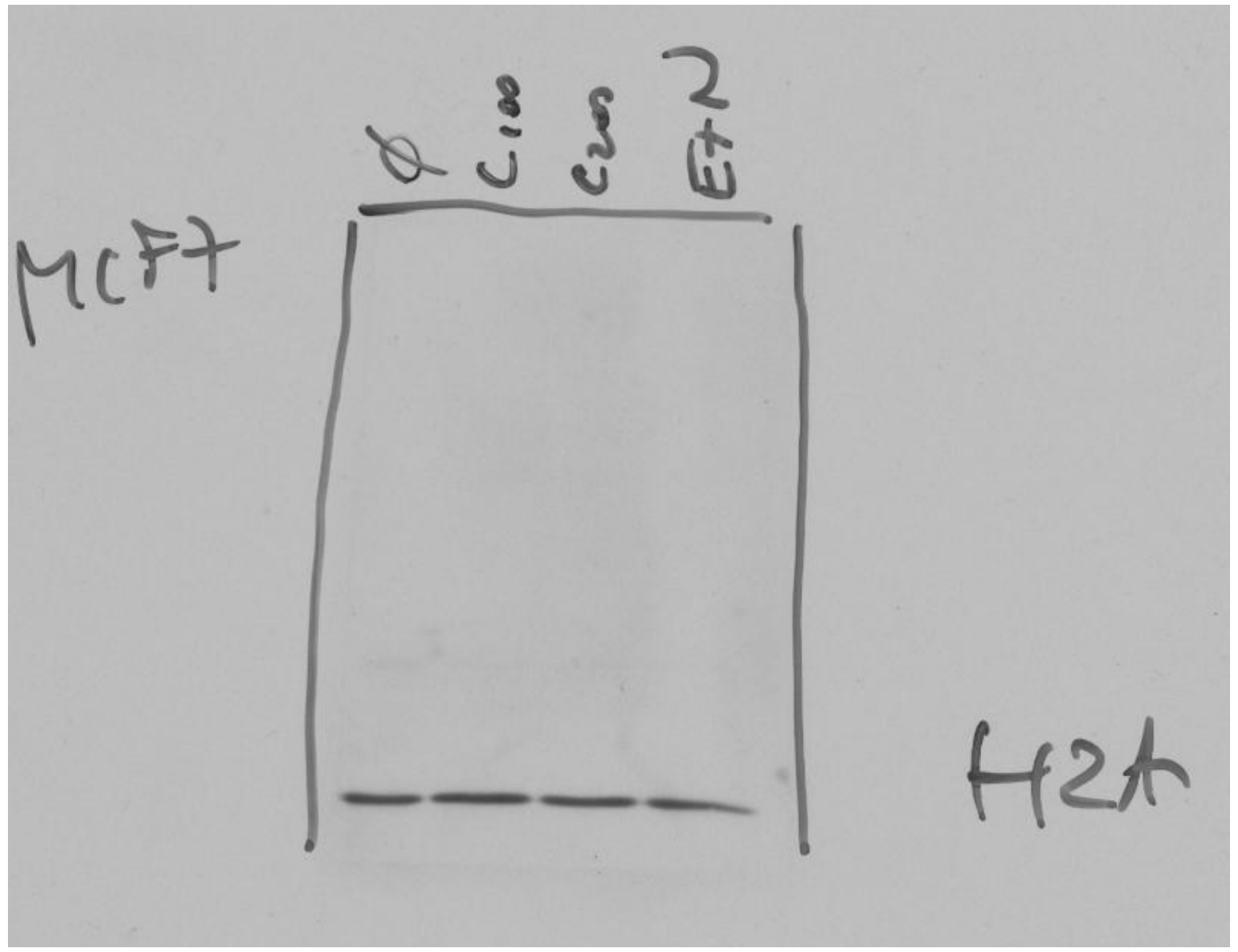
MCF7
Ø
C100
C200
E+2
H2A

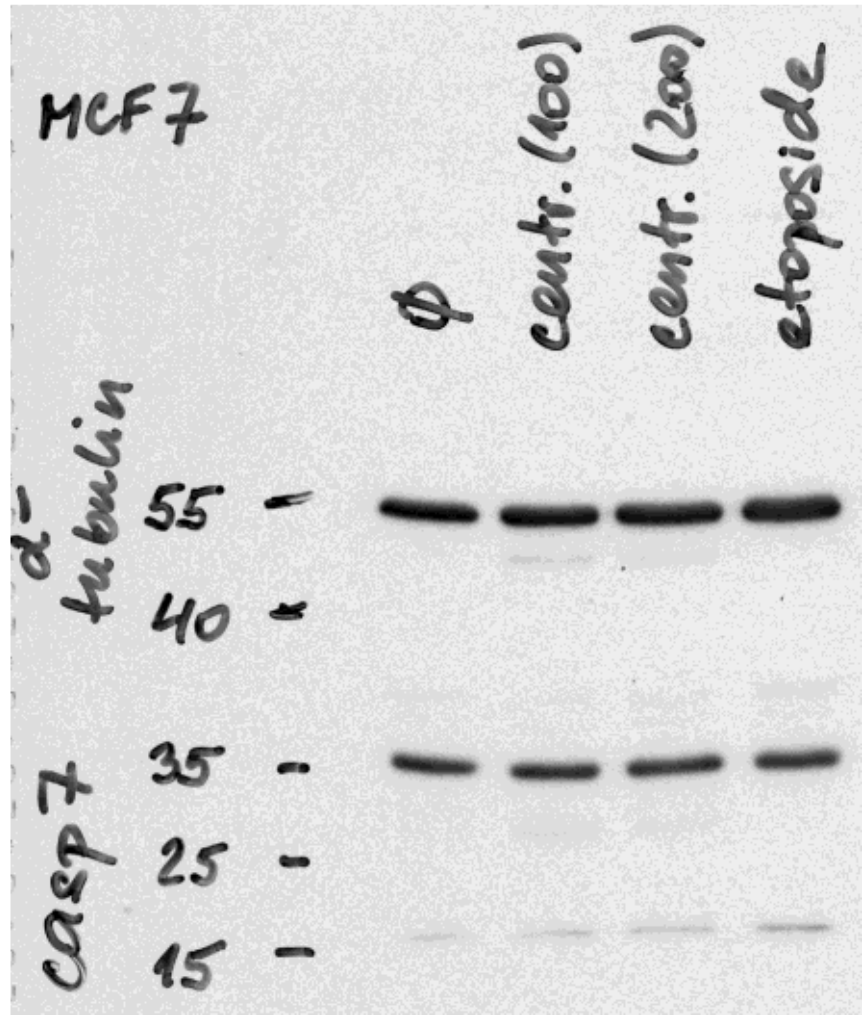
MCF7
Ø
centr. (100)
centr. (200)
etoposide
α-tubulin
55
40
casp7
35
25
15

Fig.2B

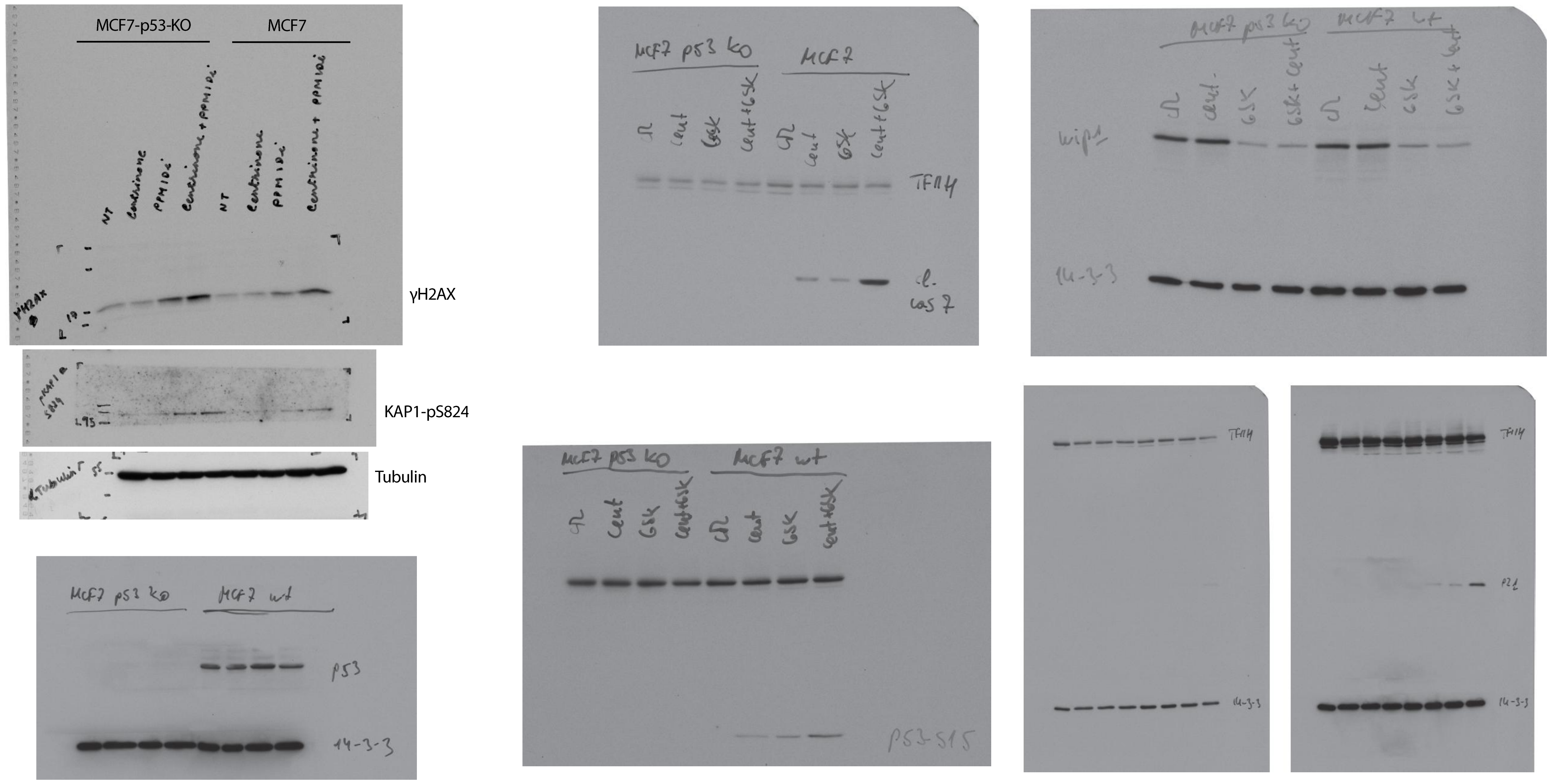

Fig.2D

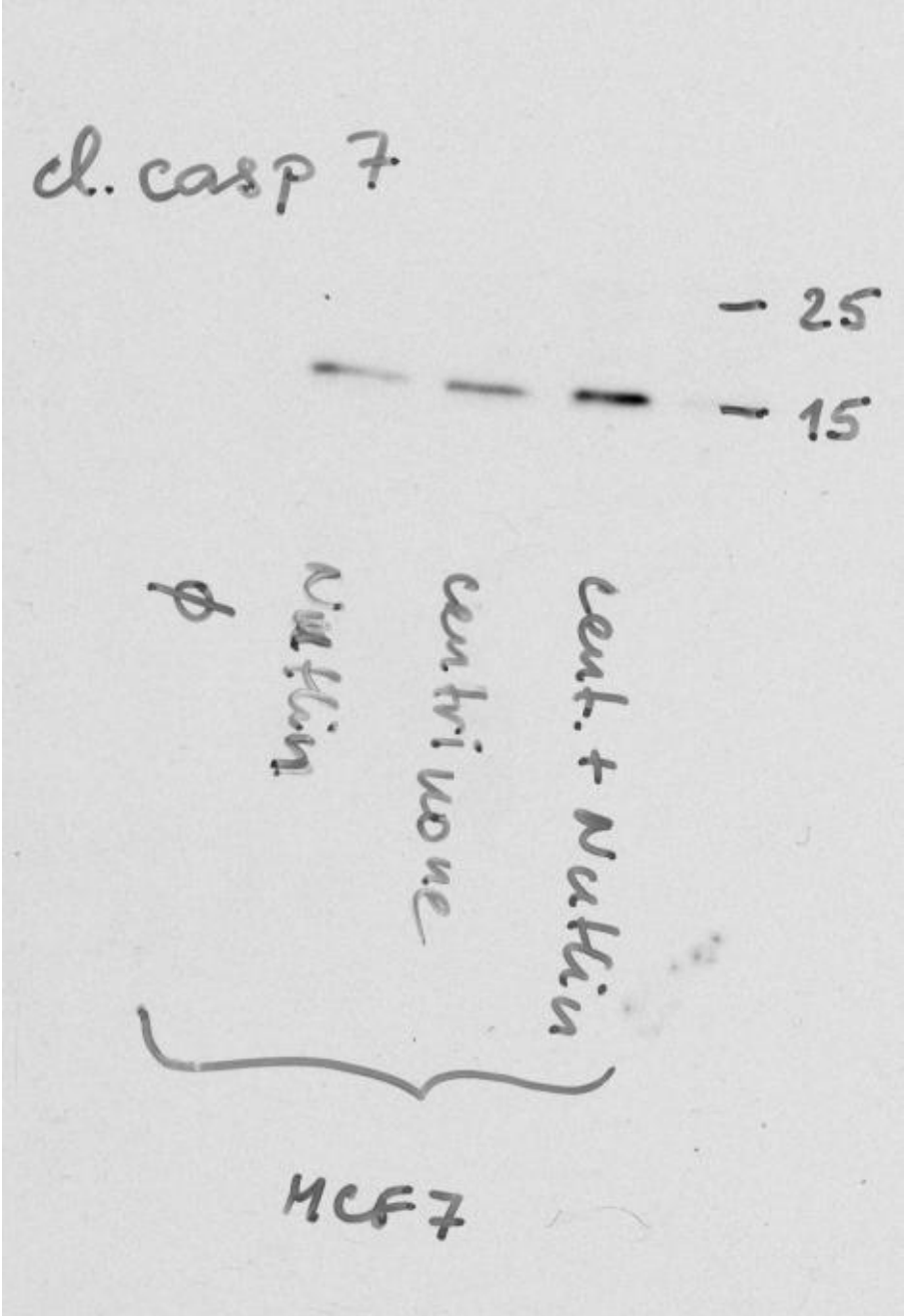

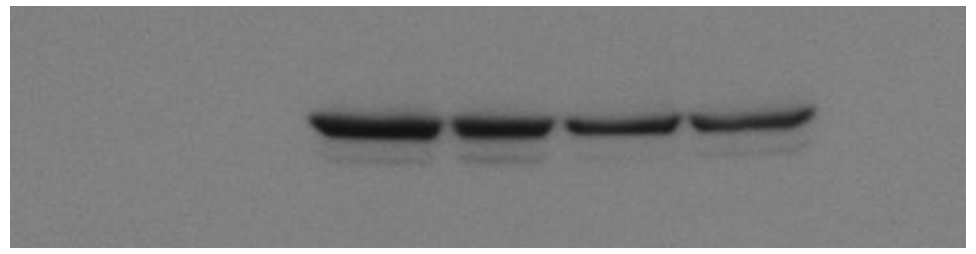

TFIIH

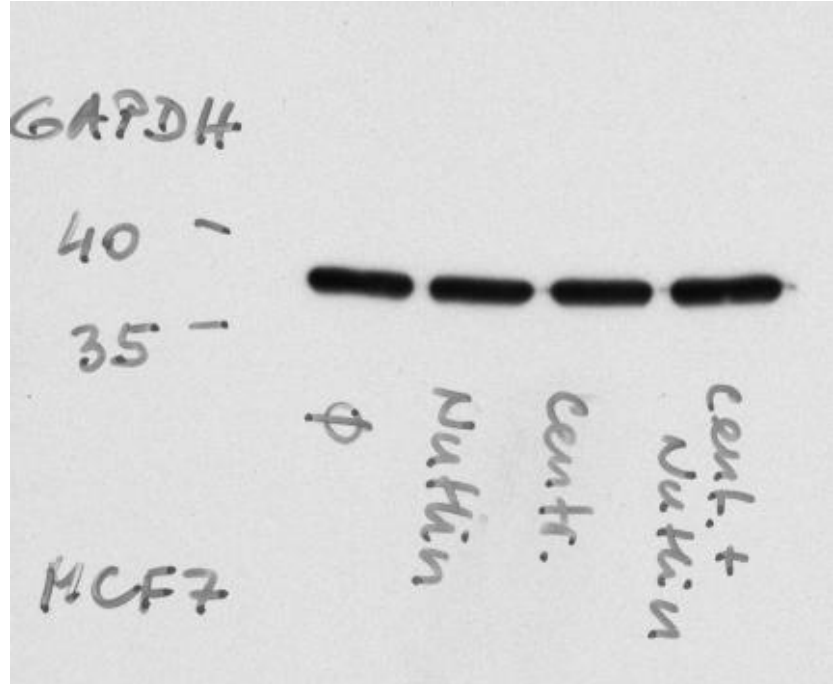

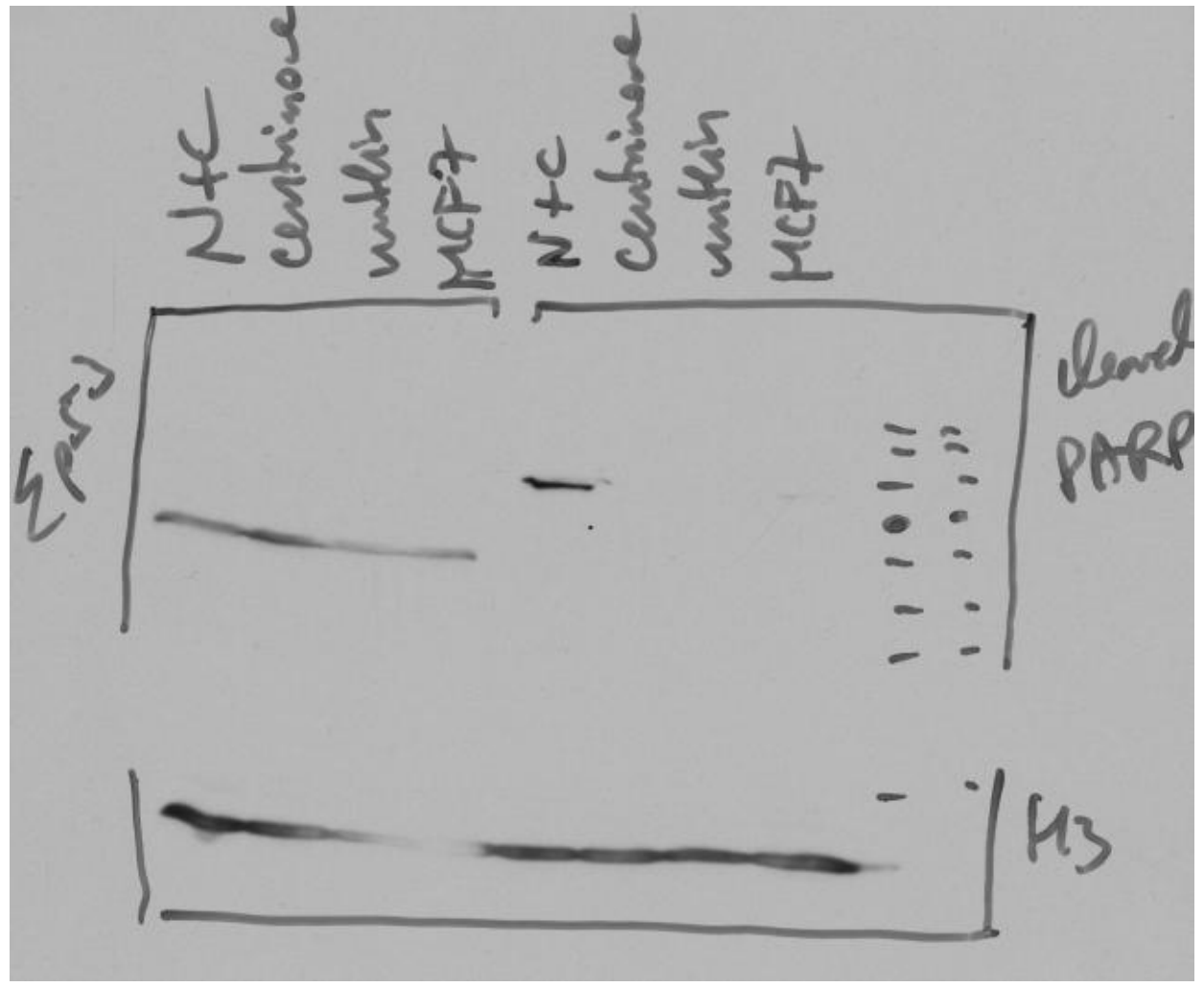

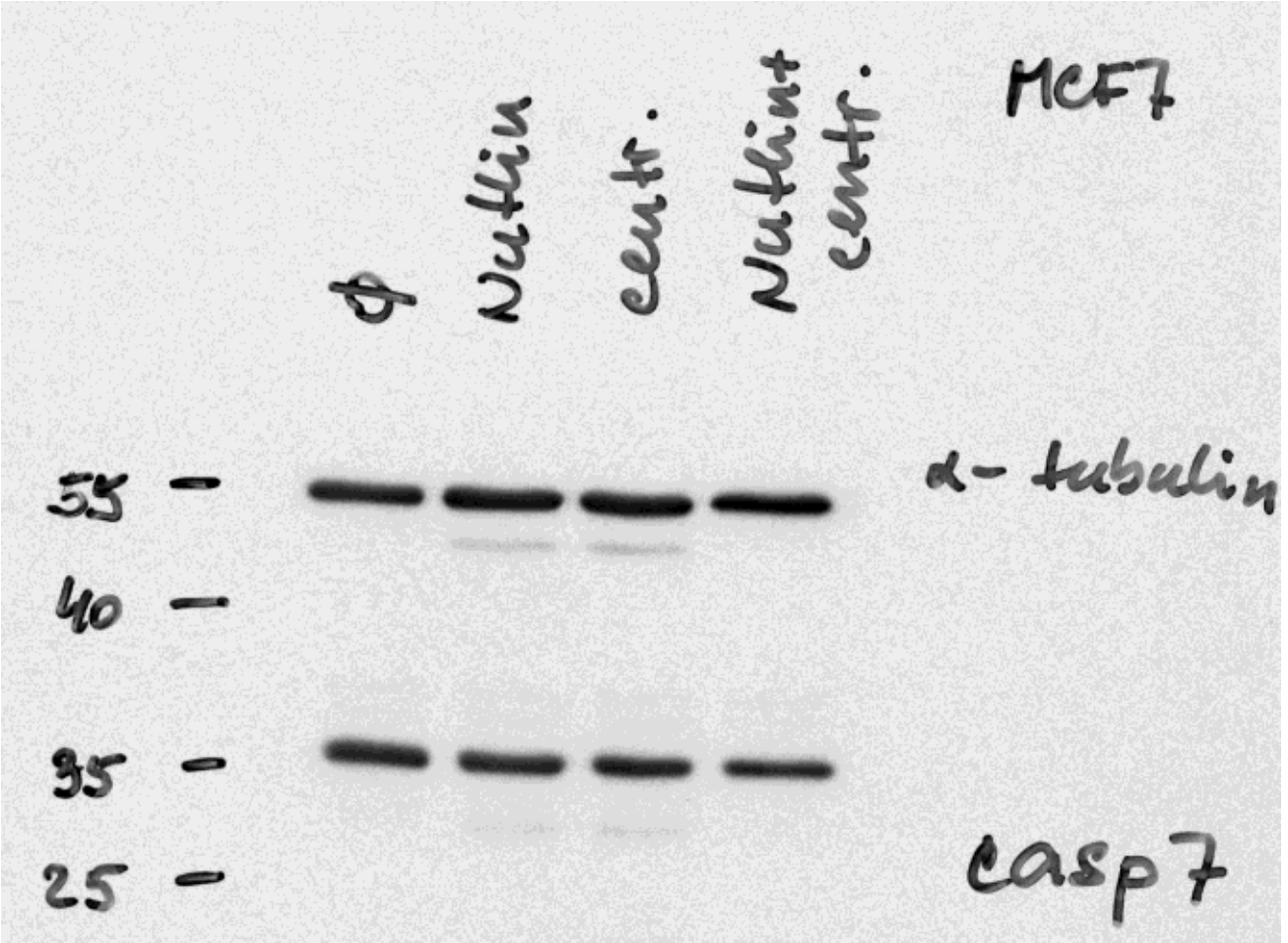

Fig. 2B continued

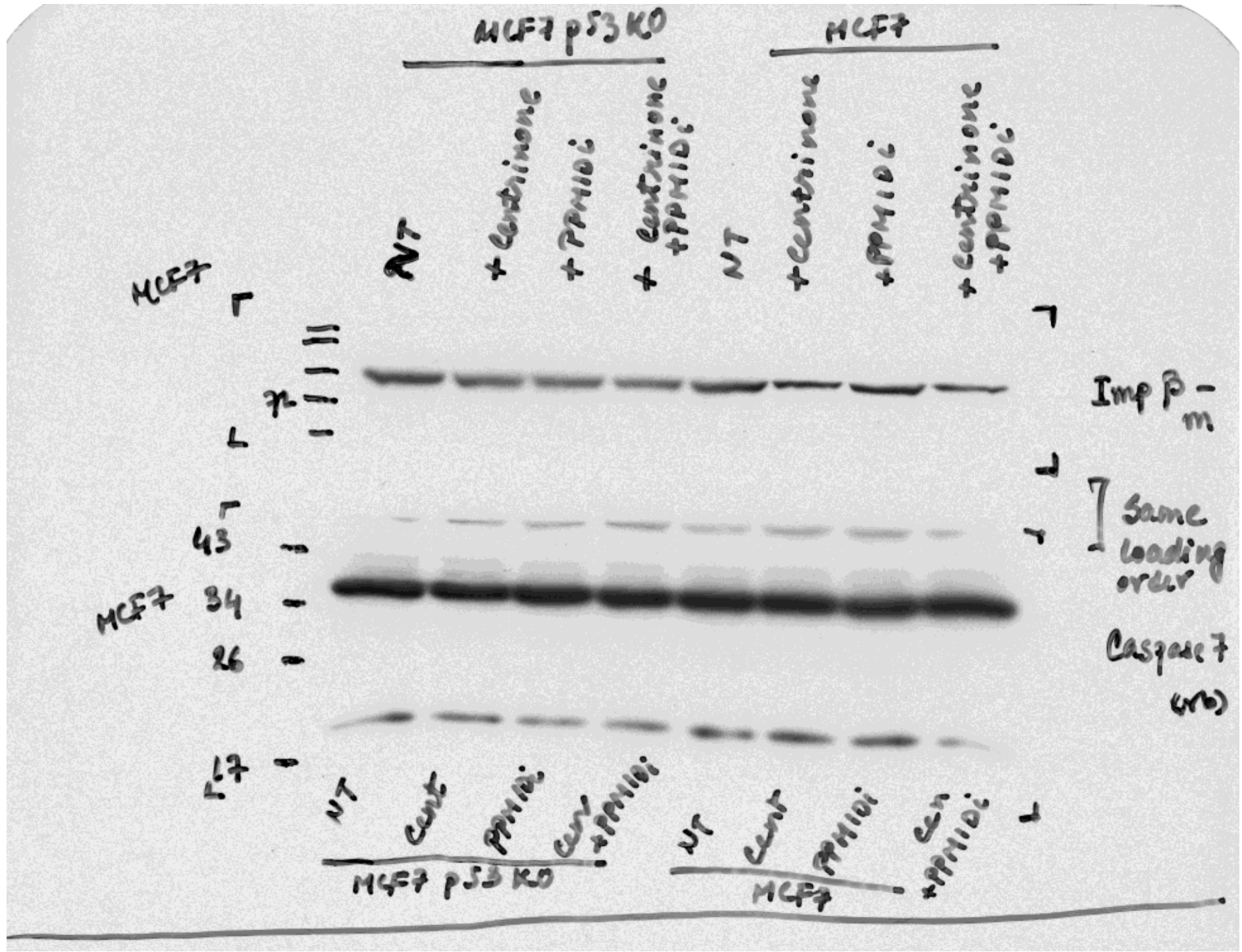

Fig. 3B

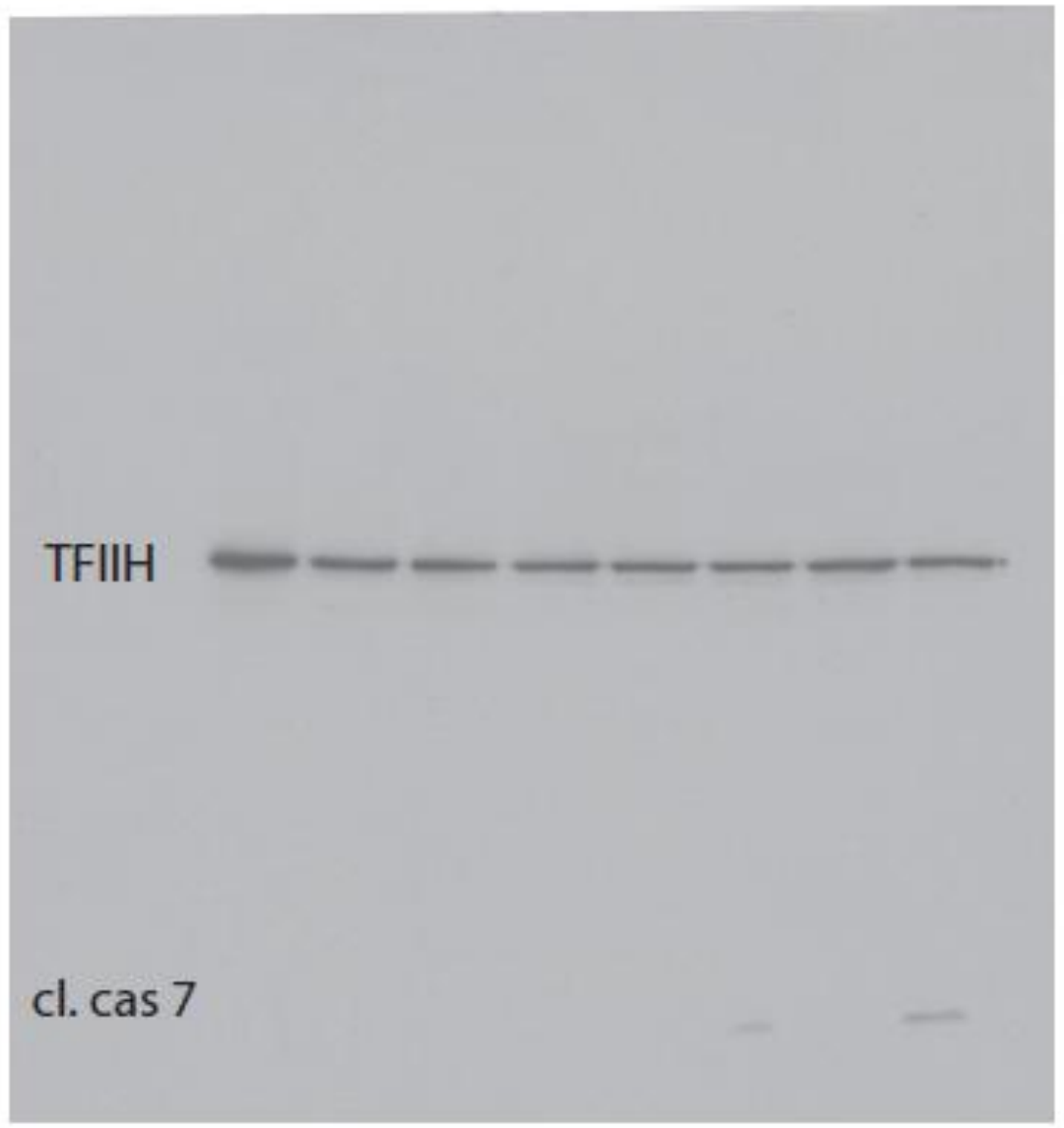

Ocifisertib in MCF7 p53-KO and MCF7 WT
(low exposure)

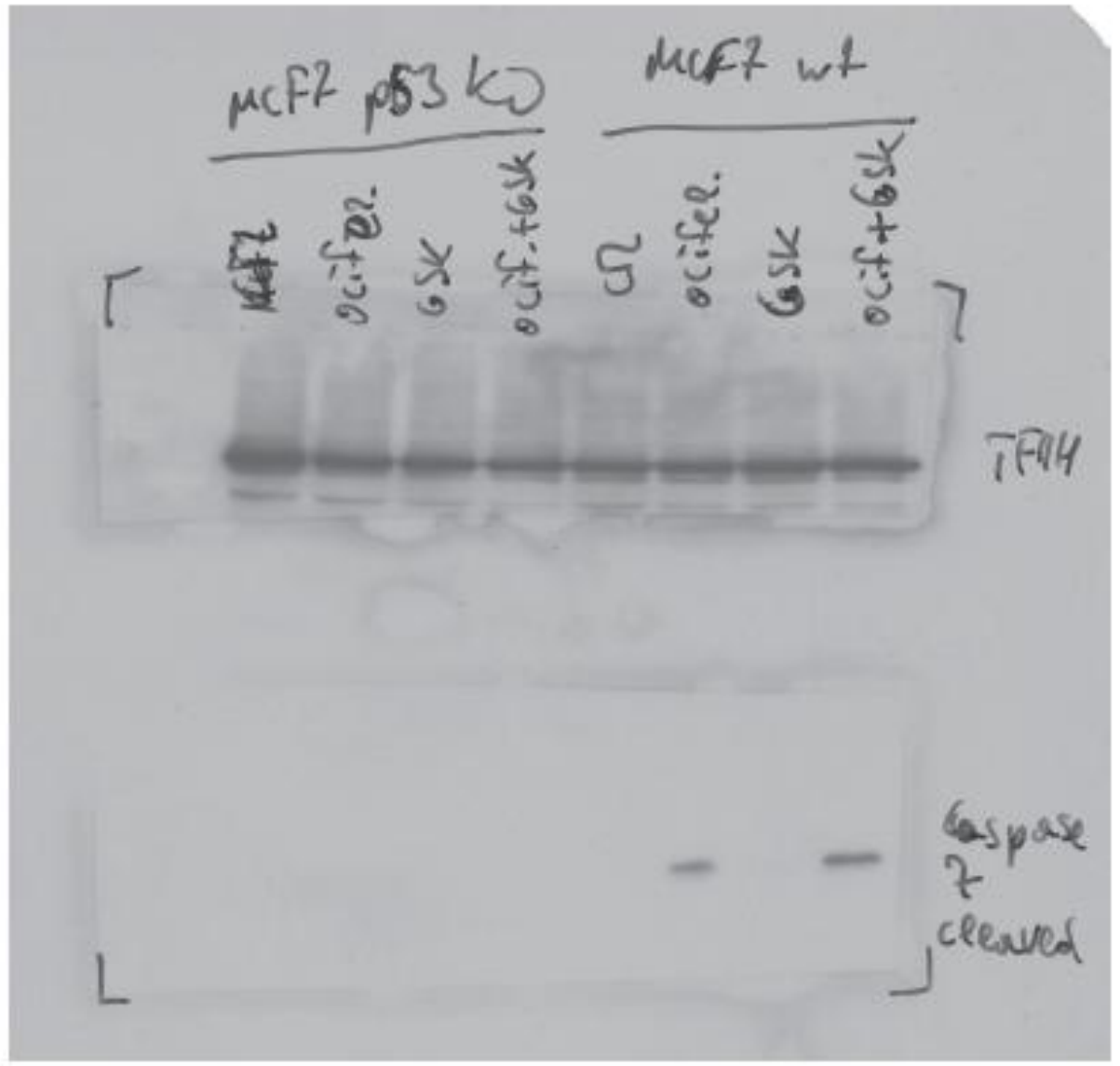

Ocifisertib in MCF7 p53-KO and MCF7 WT
(lhigh exposure)

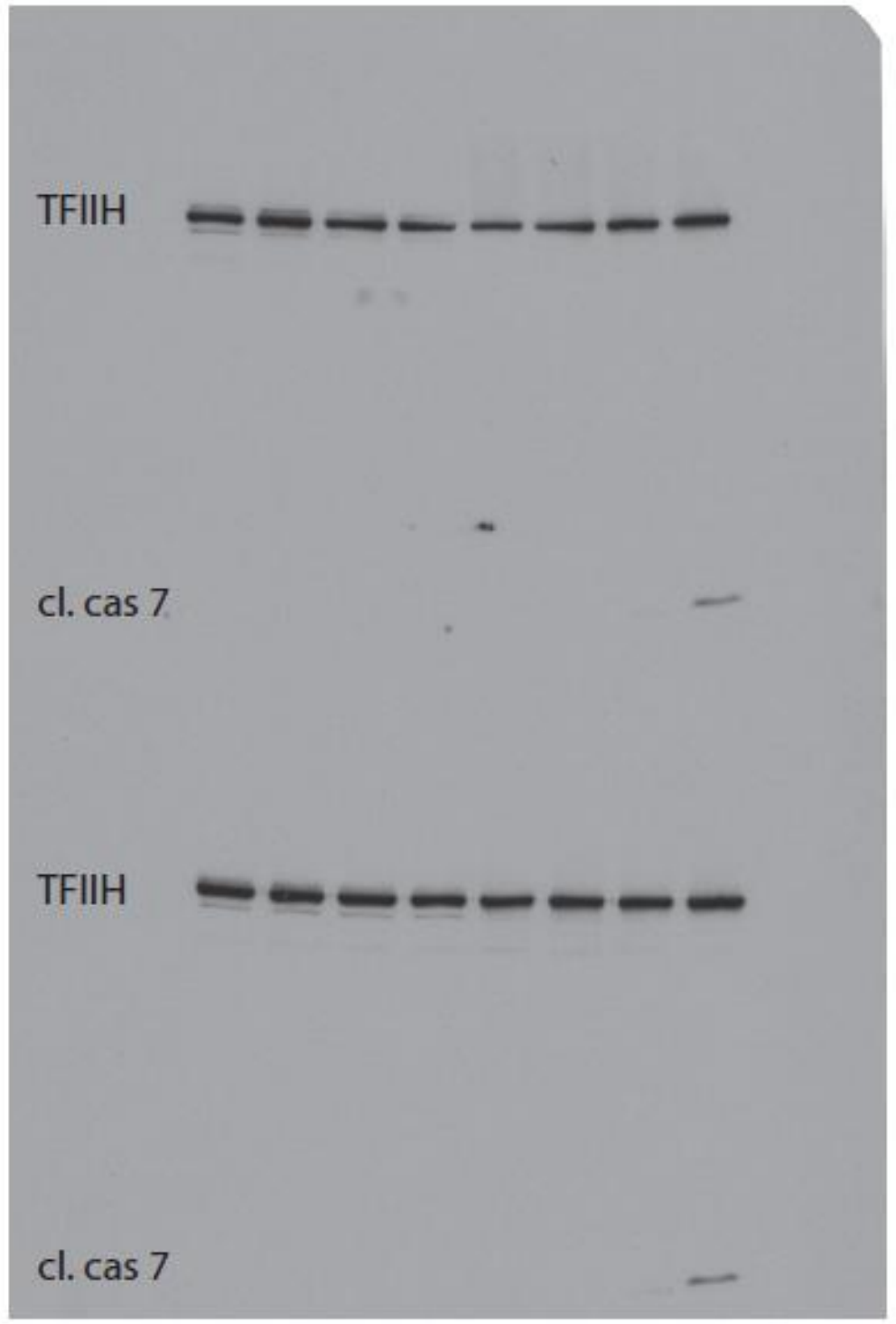

Ocifisertib in RPE and MCF7
(low exposure)

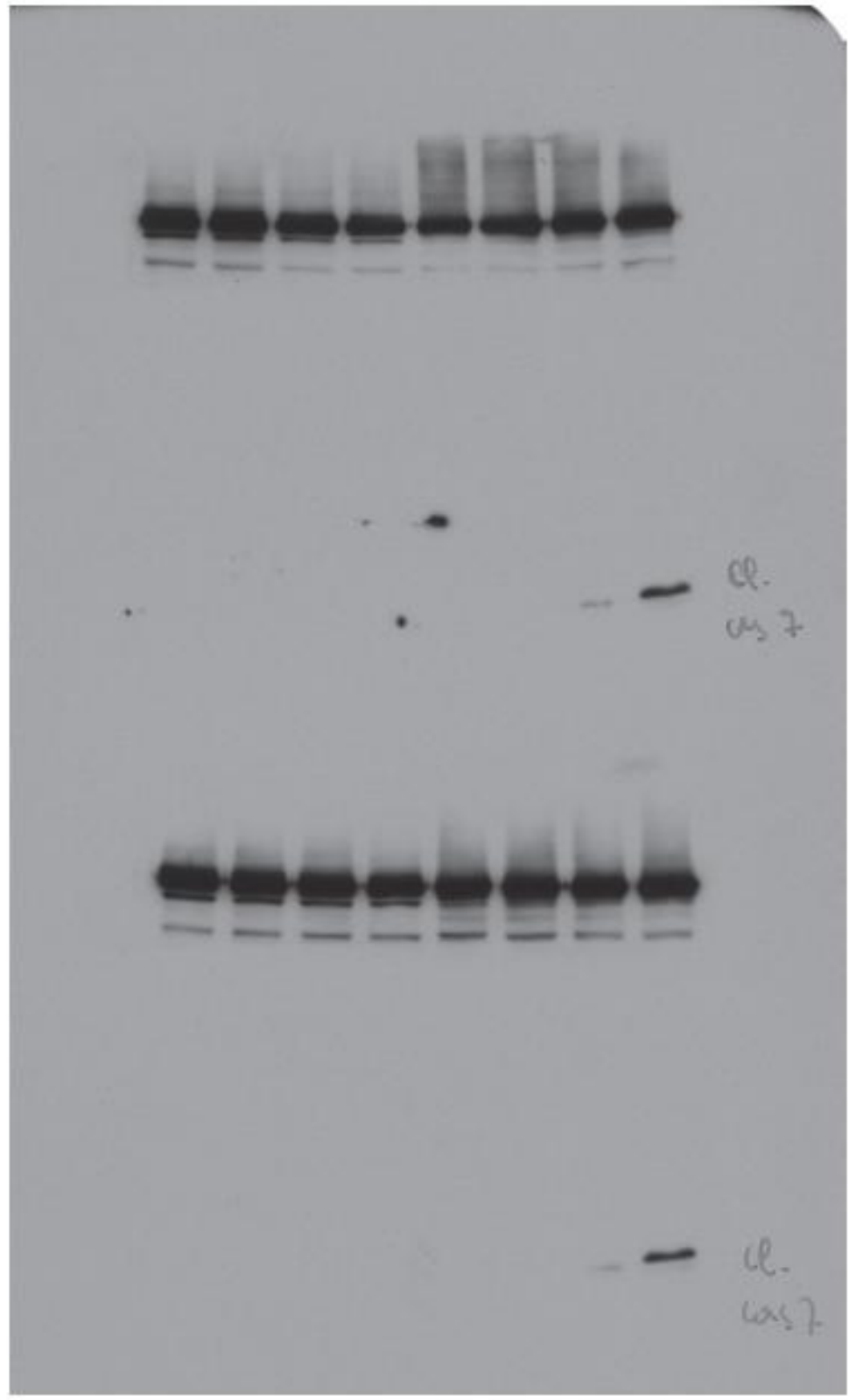

Ocifisertib in RPE and MCF7
(high exposure)

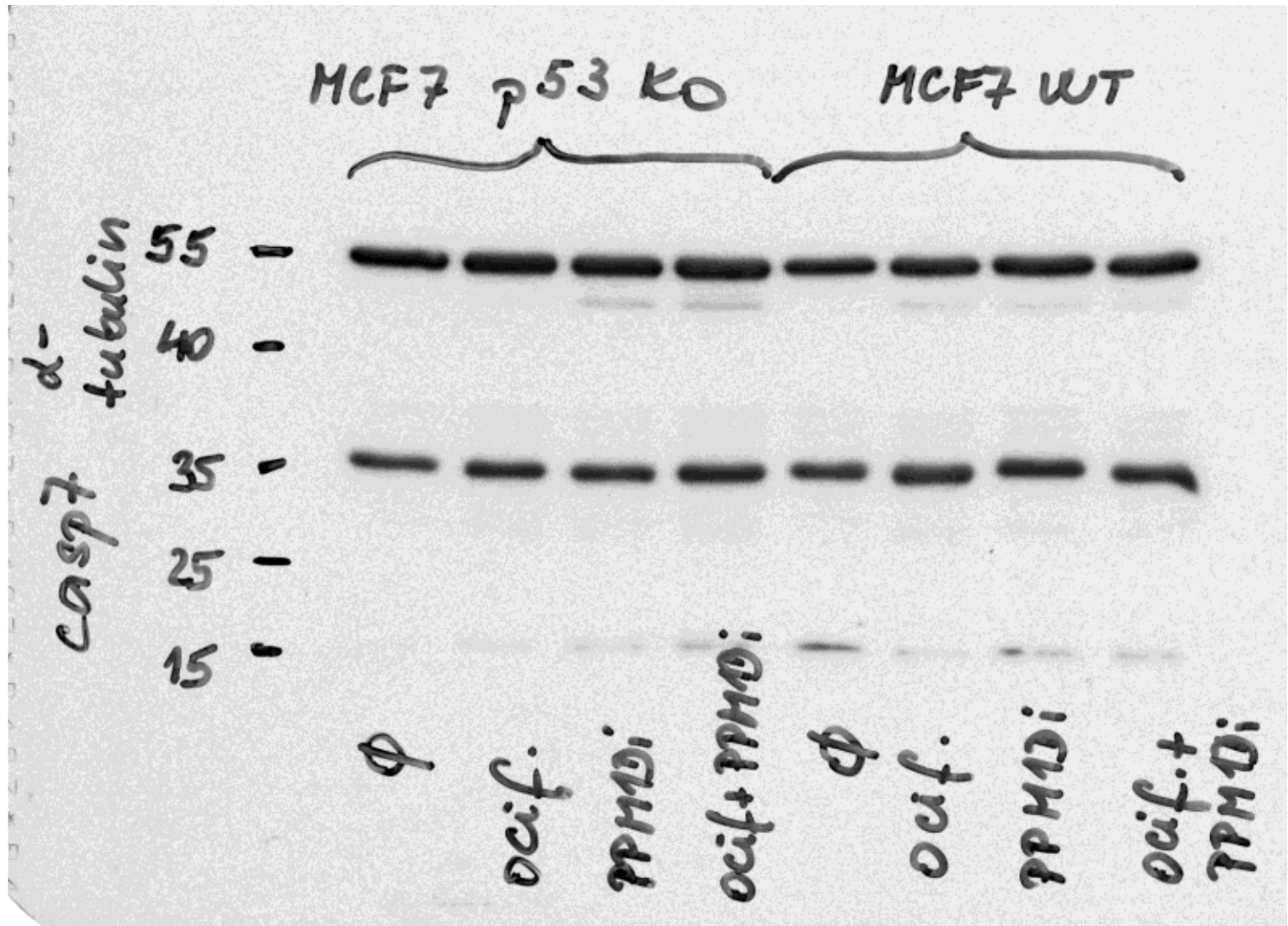

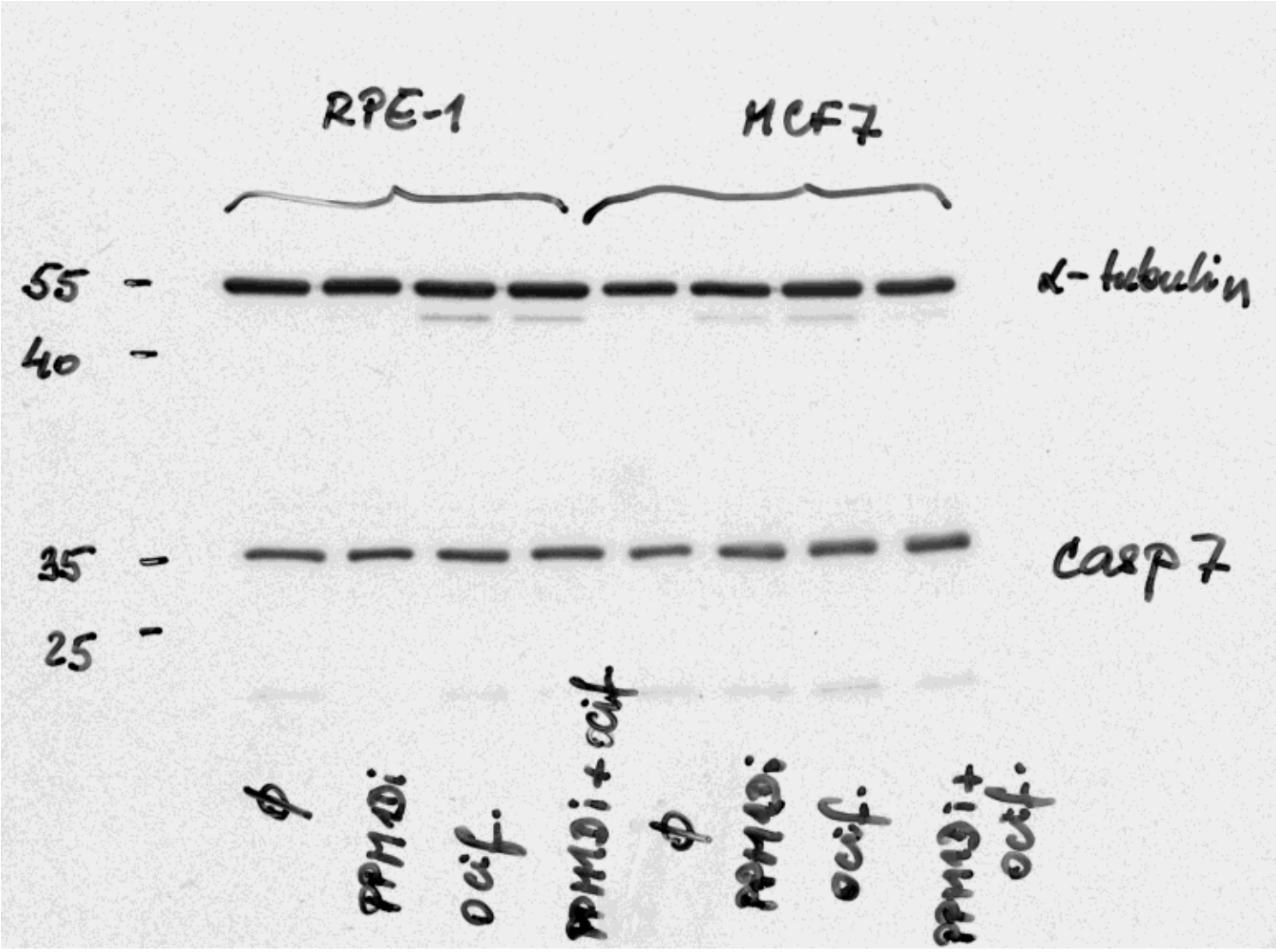

Fig. 4A

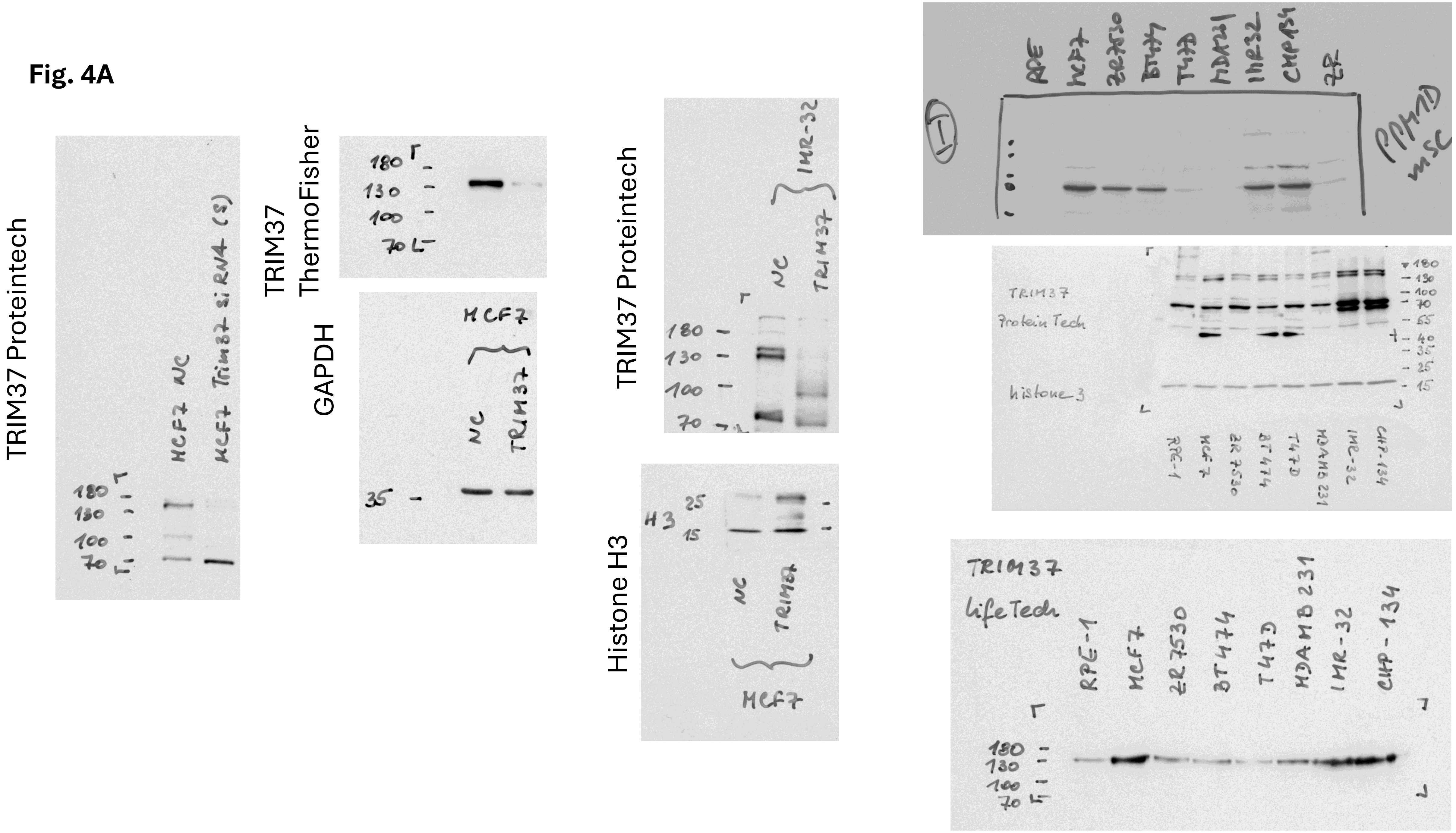
TRIM37 Proteintech
MCF7 NC
MCF7 Trim37 siRNA (s)
180
130
100
70
TRIM37 ThermoFisher
GAPDH
MCF7
NC
TRIM37
35
TRIM37 Proteintech
IMR-32
NC
TRIM37
Histone H3
H3
25
15
MCF7
RPE
MCF7
ZR7530
BT474
T47D
MDAMB231
IMR32
CHP134
ZR
TRIM37
Protein Tech
histone 3
RPE-1
MCF7
ZR 7530
BT474
T47D
MDAMB 231
IMR-32
CHP-134
65
40
35
25
TRIM37
lifeTech
RPE-1
MCF7
ZR 7530
BT474
T47D
MDAMB 231
IMR-32
CHP-134

Fig. 4B

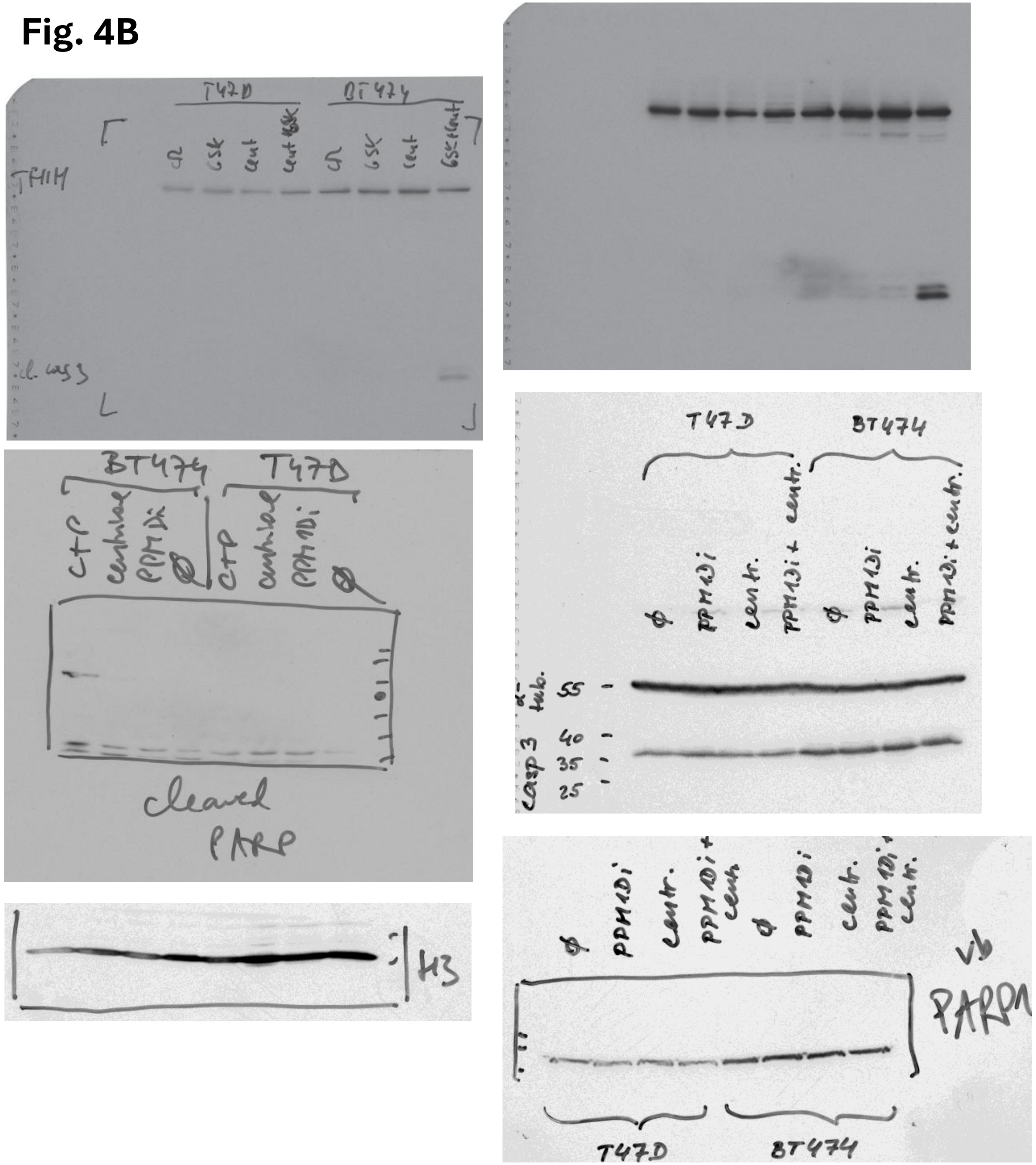
T47D
BT474
cleaved PARP
H3
PPM1Di
centr.
55
40
35
25
casp 3
PARP1

Fig. 4C

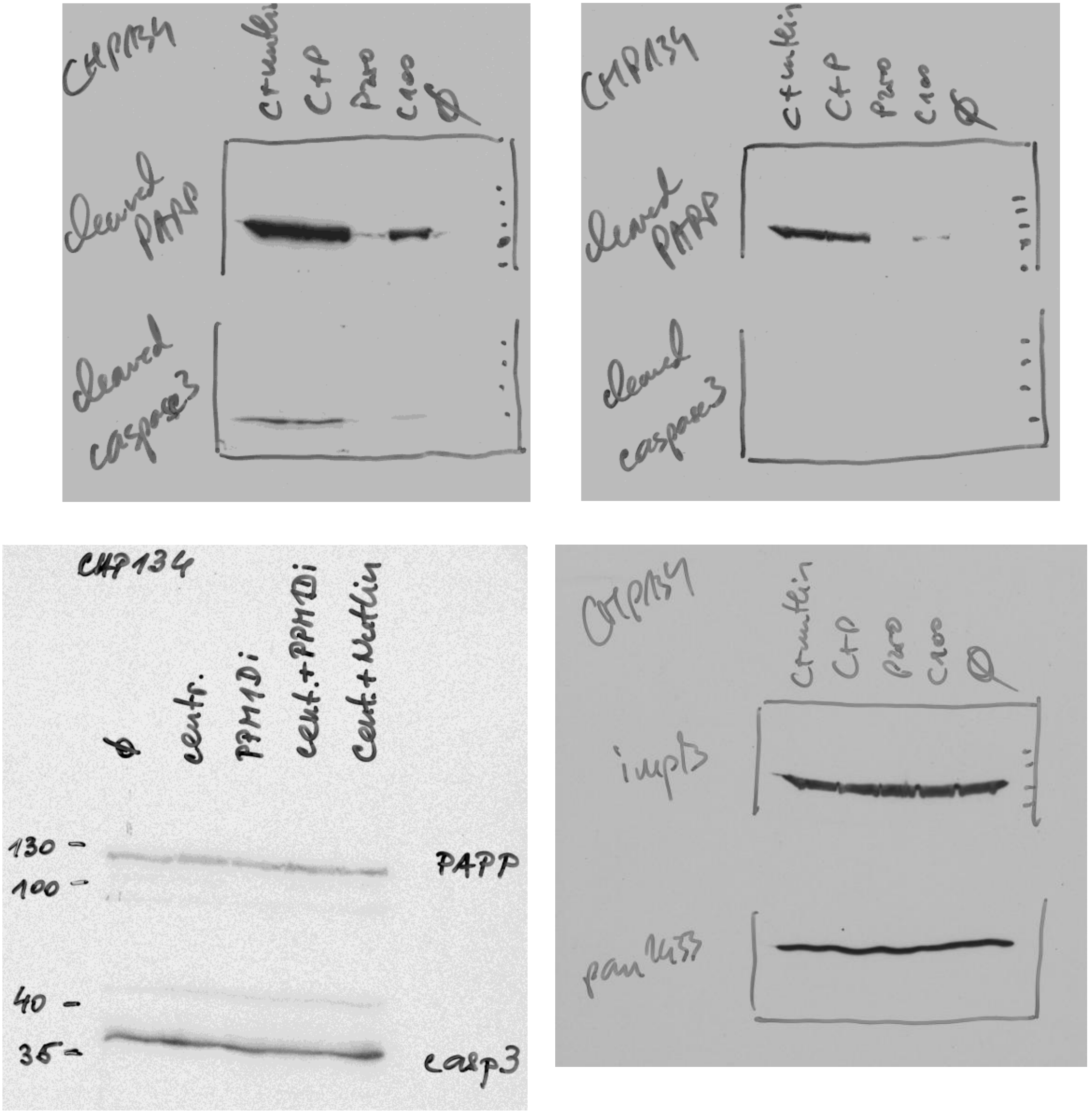
cleaved PARP
cleaved caspase3
CHP134
centr.
PPM1Di
cent.+PPM1Di
130
100
40
35
PARP
casp3

Suppl Fig 1

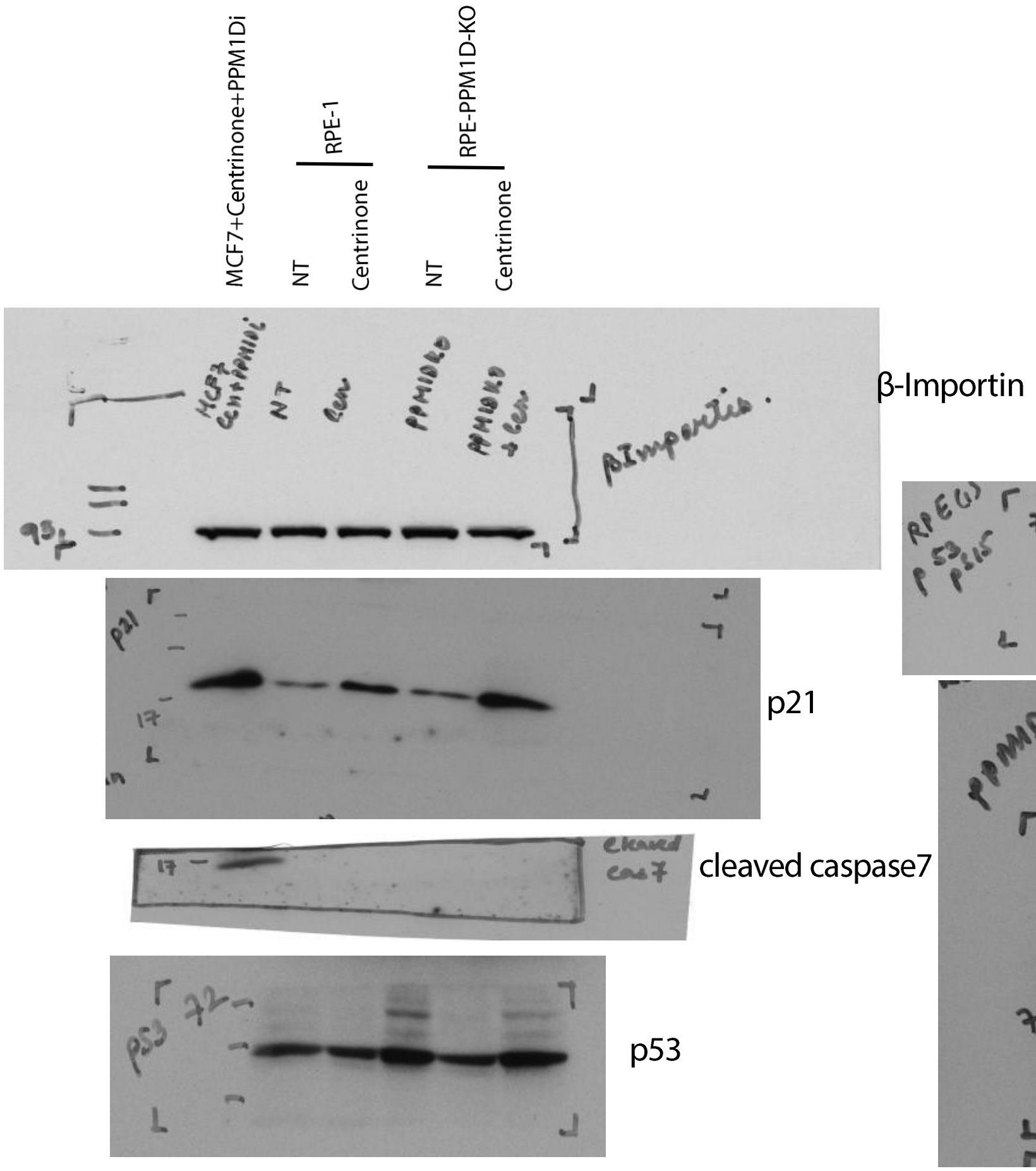
MCF7+Centrinone+PPM1Di
RPE-1
NT
Centrinone
RPE-PPM1D-KO
NT
Centrinone
β-Importin
p21
cleaved caspase7
p53

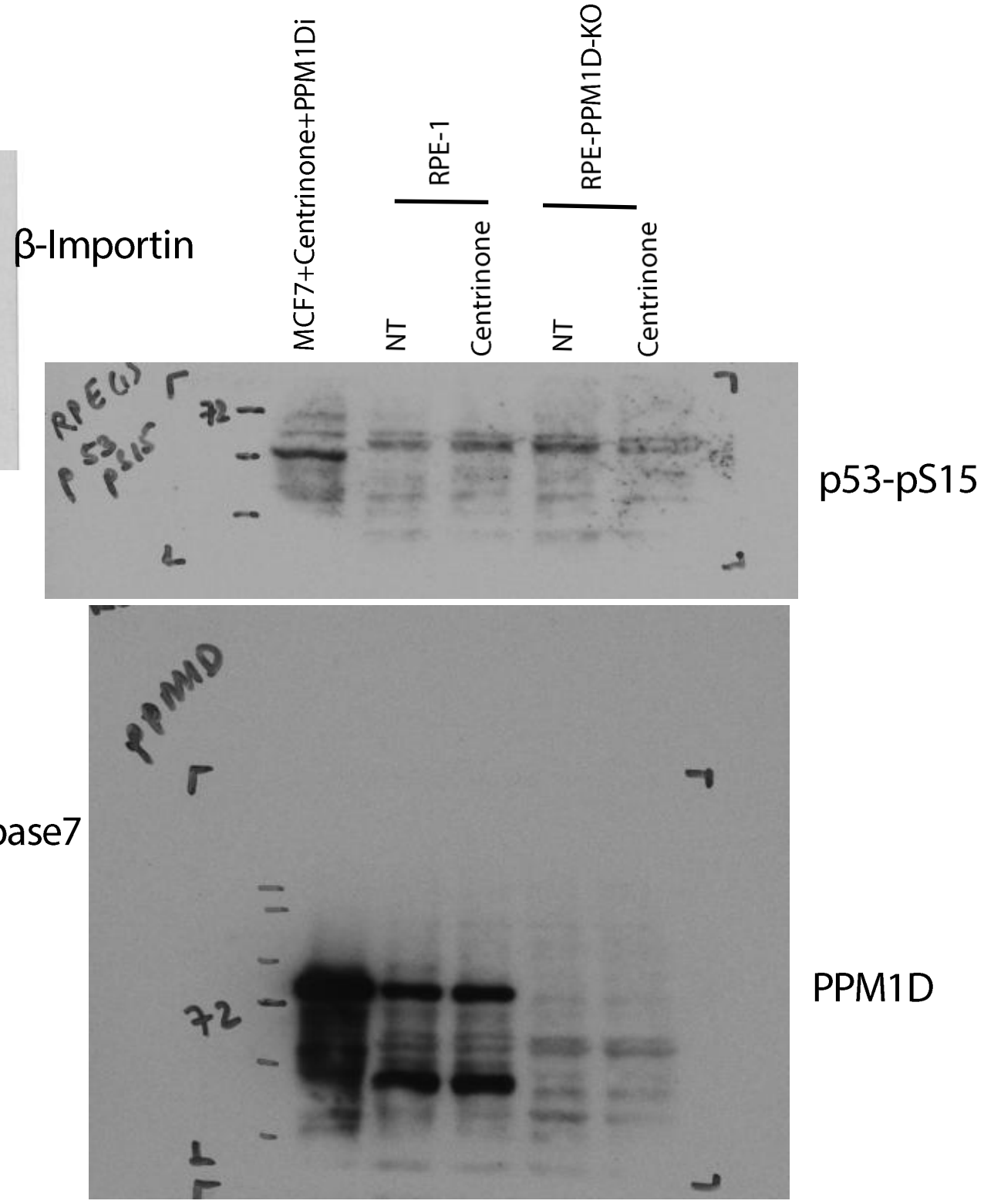
MCF7+Centrinone+PPM1Di
RPE-1
NT
Centrinone
RPE-PPM1D-KO
NT
Centrinone
p53-pS15
PPM1D
72

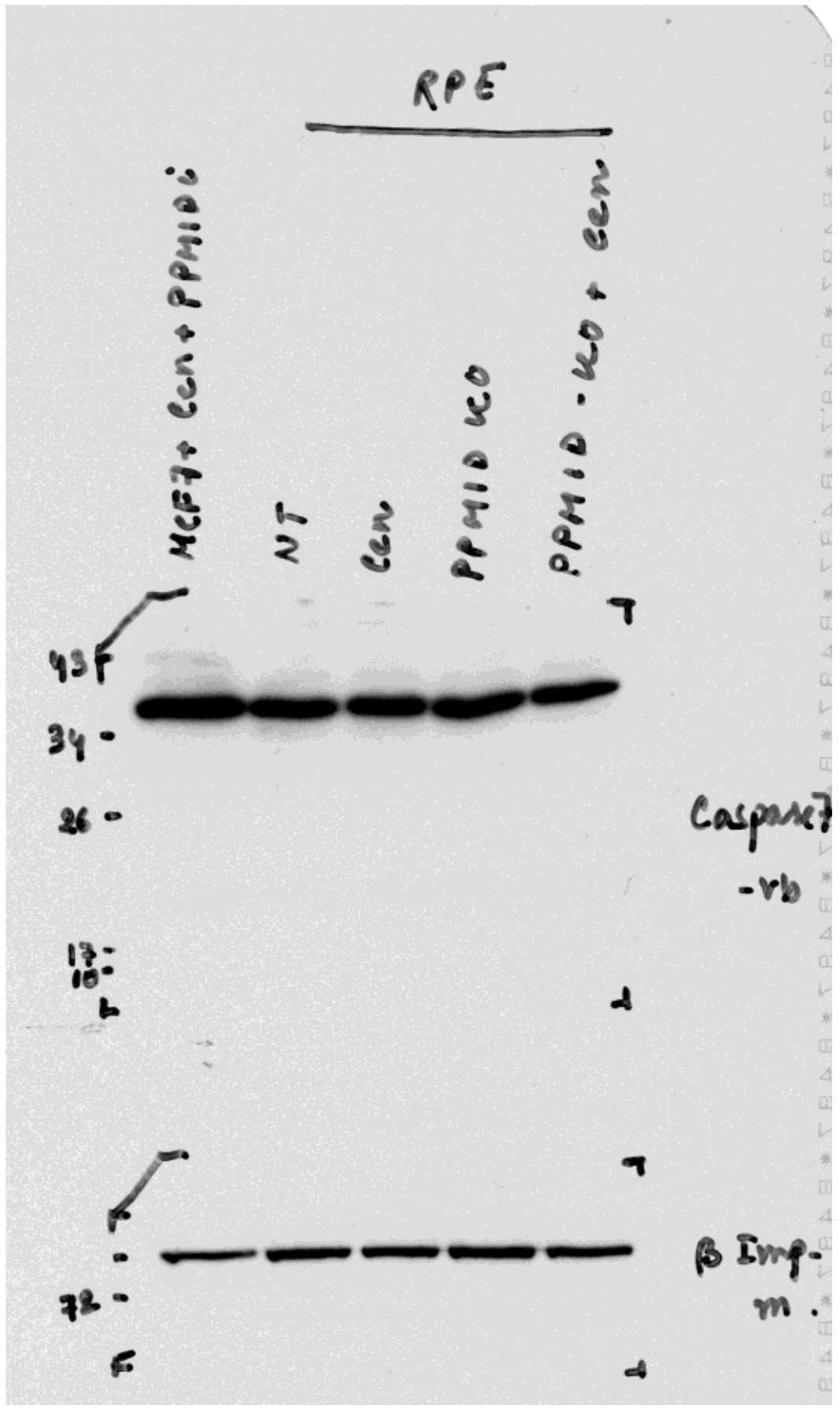
RPE
MCF7+ Cen + PPM1Di
NT
Cen
PPM1D KO
PPM1D - KO + Cen
Caspase7
-rb
β Imp-
m.

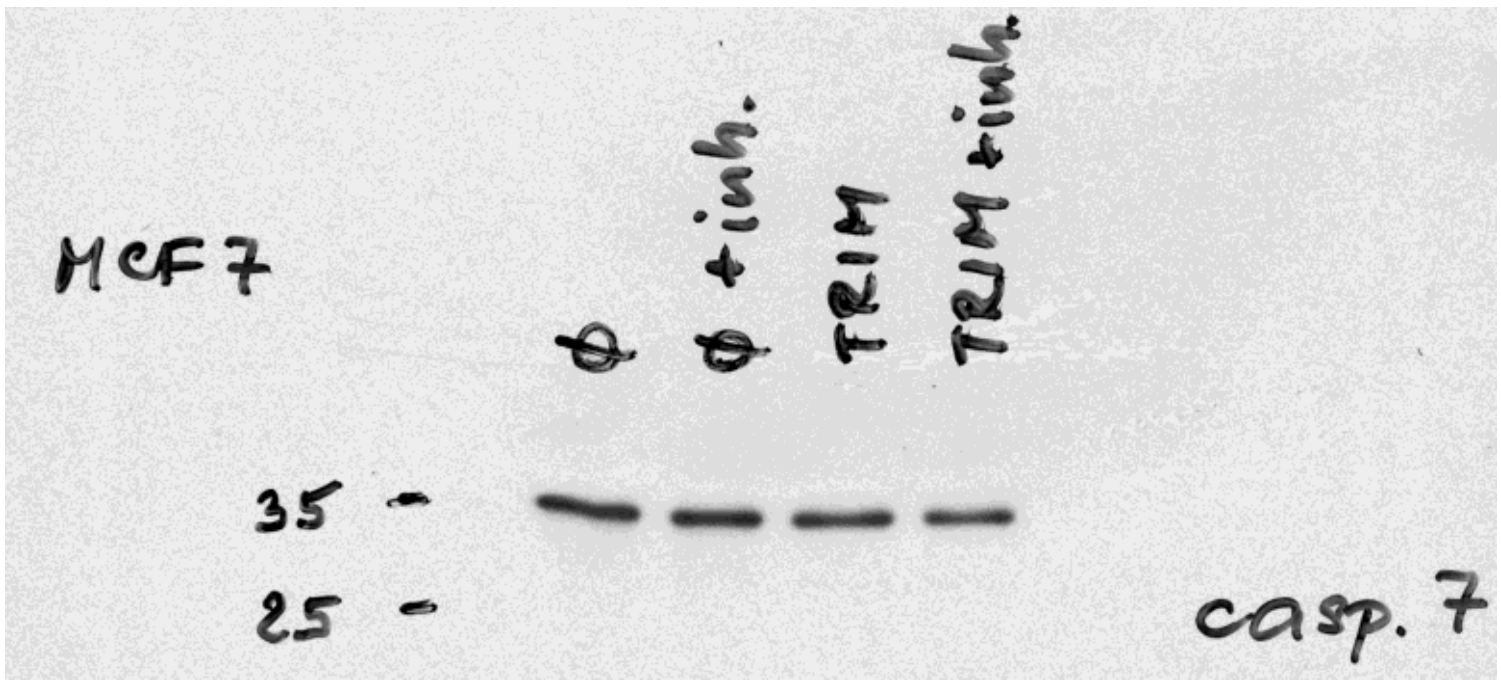
MCF7
TRIM
TRIM + inh.
35
25
casp. 7
